# Salt-inducible kinases transduce mechanical forces into the specification of the pancreatic endocrine lineage

**DOI:** 10.1016/j.stemcr.2025.102444

**Published:** 2025-03-06

**Authors:** Chenglei Tian, Adam Rump, Christine Ebeid, Anant Mamidi, Henrik Semb

**Affiliations:** 1 Institute of Translational Stem Cell Research, Helmholtz Diabetes Center, Helmholtz Zentrum Munchen, Munich, Germany; 2 Novo Nordisk Foundation Center for Stem Cell Biology (DanStem), University of Copenhagen, Copenhagen, Denmark

**Keywords:** salt-inducible kinases, YAP1, bipotent pancreatic progenitors, endocrine progenitors, mechanotransduction, beta cell

## Abstract

The extracellular matrix-F-actin-Yes-associated protein 1 (YAP1)-Notch mechanosignaling axis is a gatekeeper in the fate decisions of bipotent pancreatic progenitors (bi-PPs). However, the link between F-actin dynamics and YAP1 activity remains poorly understood. Here, we identify salt-inducible kinases (SIKs) as mediators of F-actin-triggered changes in YAP1 activity. Interestingly, sodium chloride treatment promotes the differentiation of bi-PPs into NEUROG3^+^ endocrine progenitors (EPs) through enhanced SIK expression. Consistently, the pan-SIK inhibitor HG-9-09-01 (HG) inhibits latrunculin B (LatB)-induced EP differentiation via nuclear YAP1 accumulation. Unexpectedly, withdrawal of HG after a 12-h treatment increased SIK expression by a negative feedback mechanism, leading to significantly enhanced endocrinogenesis. Therefore, the combined treatment of bi-PPs with LatB and HG for 12 h boosted endocrinogenesis, ultimately leading to an increased number of beta cells. In summary, we identify SIKs as new transducers of mechanotransduction-triggered induction of pancreatic endocrine cell fates.

## Highlights

• SIKs act as the link between F-actin and YAP1 during endocrinogenesis
• Increased expression of SIKs promotes endocrine progenitor differentiation
• SIK inhibition reduces endocrine progenitor differentiation

## Graphical abstract

**Figure:**
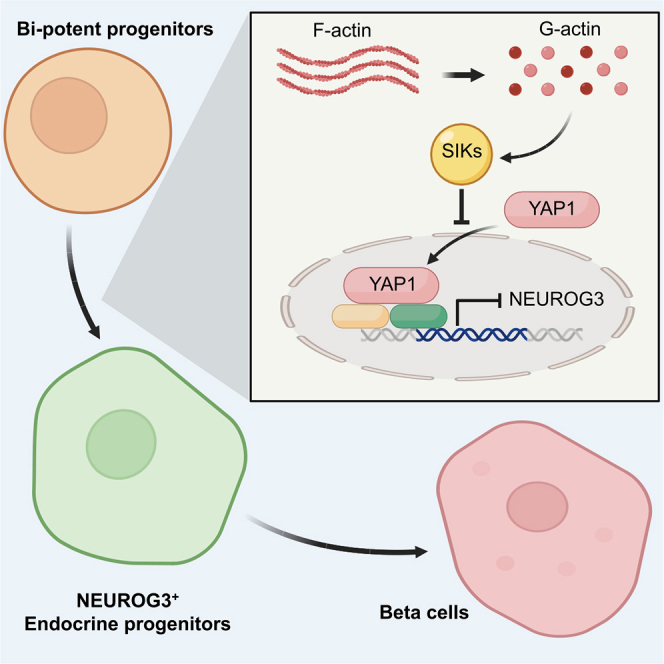

## Teaser

Semb and colleagues identify SIKs as new transducers of mechanotransduction-triggered induction of pancreatic endocrine cell fates. Pharmacologically increasing SIK expression/activity enhances endocrinogenesis and beta cell differentiation.

## Introduction

Type 1 diabetes (T1D) is an autoimmune disease in which beta cells are selectively destroyed, leading to severe insulin deficiency that requires daily insulin injections for survival (Atkinson et al., 2014). Theoretically, human pluripotent stem cells (hPSCs) serve as an unlimited source of functional beta cells, offering a promising advancement in therapy for the growing number of patients with T1D (Schroeder, 2012). The differentiation of beta cells from hPSCs relies on the precise activation and repression of specific developmental pathways by recombinant proteins and chemicals, guiding hPSCs stepwise through stages such as definitive endoderm, primitive gut tube, posterior foregut, bi-potent pancreatic progenitor (bi-PP), endocrine progenitor (EP), and endocrine cell stages (Pagliuca et al., 2014; Rezania et al., 2014; Russ et al., 2015). Among these stages, PDX1^+^NKX6.1^+^ bi-PPs have the potential to differentiate into either ductal or endocrine cell lineages. While most established protocols can consistently and efficiently produce bi-PPs *in vitro*, the efficiency of further differentiation from bi-PPs to EPs is imperfect due to lack of full mechanistic comprehension of how this lineage decision is controlled (Ameri et al., 2017; Hogrebe et al., 2020; Mamidi et al., 2018; Pagliuca et al., 2014; Rezania et al., 2014; Russ et al., 2015).

We previously demonstrated that F-actin dynamics, e.g., in response to cell-extracellular matrix (ECM) adhesion, autonomously controls the fate of bi-PPs through Yes-associated protein 1 (YAP1)-Notch mechanosignaling. Specifically, actin depolymerization decreases nuclear YAP1 levels, leading to reduced HES1 expression and increased EP differentiation (Mamidi et al., 2018). Consistent with our findings, Hogrebe et al. reduced F-actin levels in stem cell-derived bi-PPs by latrunculin A treatment, thereby promoting endocrinogenesis and beta cell formation (Hogrebe et al., 2020; Siehler et al., 2020). However, the mechanism by which F-actin regulates nuclear YAP1 activity in bi-PPs remains unknown. Here, we identify salt-inducible kinases (SIKs), members of the AMP-activated protein kinase family, as mediators of the F-actin-triggered effects on YAP1 activity during lineage specification of bi-PPs (Wang et al., 2020). Reduction of F-actin levels by latrunculin B (LatB) increases SIK expression, which diminishes nuclear levels of YAP1 and increases EP differentiation. Consistently, pharmacologically increased SIK expression promotes endocrine cell differentiation and generates more beta cells both *in vitro* (hPSC differentiation) and *ex vivo* (mouse fetal explants). Altogether, these studies reveal the role of SIKs in controlling the fate decision of bi-PPs and provide a valuable new way to increase the conversion of hPSC-derived bi-PPs into endocrine cells.

## Results

### The pro-endocrinogenic effect of actin depolymerization is mediated by SIKs

**Figure 1:**
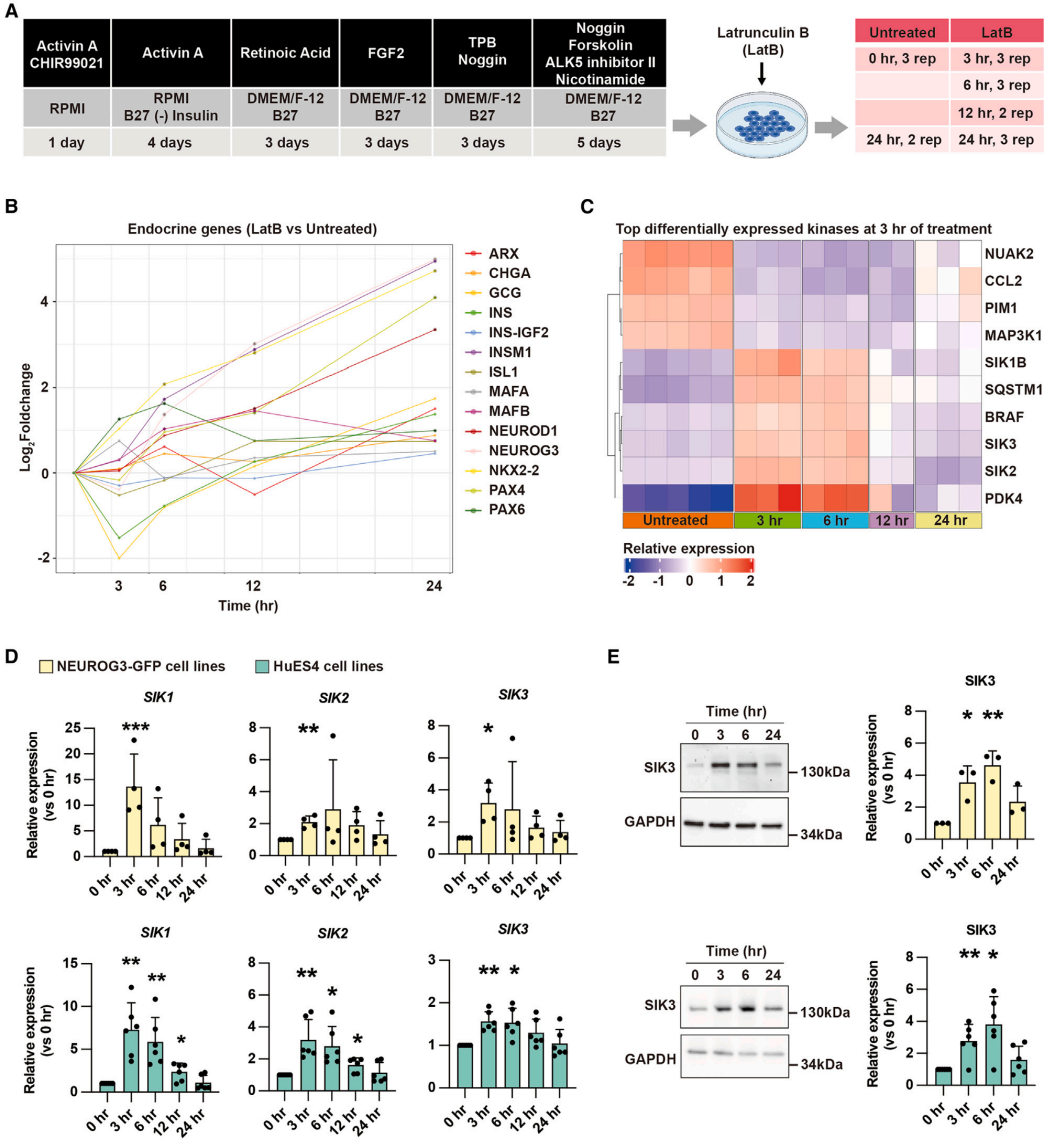
Salt-inducible kinases are induced by latrunculin B in pancreas progenitors (A) Experimental setup for using bulk RNA-seq to assess the potential downstream targets of F-actin in endocrine cell differentiation. The differentiation protocol generates pancreatic bipotent progenitors (bi-PPs) by day 19. At day 19, latrunculin B (LatB), an actin depolymerization drug, is added to the culture. Samples are then collected at 0, 3, 6, 12, and 24 h post treatment for bulk RNA-seq analysis. (B) RNA-seq-based time-course analysis of expression of pancreatic endocrine cell markers after LatB treatment. Most of these markers are upregulated at 6 h post LatB treatment. The samples are derived from the NEUROG3-GFP cell lines. (C) Top differentially expressed kinase-coding genes between the 3 h LatB treatment and untreated samples (relative expression >2, adjusted *p* value < 0.05). The samples are derived from the NEUROG3-GFP cell lines. (D) qPCR-based time-course analysis of *SIK1*, *SIK2*, and *SIK3* expression levels after LatB treatment. Data represent the mean ± SD. The samples are derived from both NEUROG3-GFP (*n* = 4 independent repeats) and HuES4 (*n* = 6 independent repeats) cell lines. (E) Western blot-based time-course analysis of SIK3 protein levels after LatB treatment. Data represent the mean ± SD. The samples are derived from both NEUROG3-GFP (*n* = 4 independent repeats) and HuES4 (*n* = 6 independent repeats) cell lines. ^∗^*p* < 0.05; ^∗∗^*p* < 0.01; ^∗∗∗^*p* < 0.001 (two-tailed paired Student’s t test). See also Figure S1; Table S1.

To explore the molecular mechanism by which actin depolymerization triggers endocrine cell differentiation, we treated PDX1^+^NKX6.1^+^ bi-PPs with the actin depolymerization drug LatB on day 19, as previously done (Mamidi et al., 2018) (Figures 1A, S1A, and S1B). To precisely identify the F-actin-targeted genes responsible for triggering EP differentiation, we collected samples at multiple time points (3, 6, 12, and 24 h) following LatB treatment for bulk RNA-seq analysis. Untreated samples at 0 and 24 h were used as controls (Figure 1A). Analysis of endocrine cell marker gene expression revealed that early EP marker genes, including *NEUROG3*, *INSM1*, *ISL1*, and *NEUROD1*, increased 6 h post LatB treatment (Figure 1B; Table S1). Therefore, our analysis focused on the 3-h post-LatB treatment time point.

Surprisingly, the most significantly upregulated gene (by adjusted *p* value) 3 h post LatB treatment was the protein kinase *SIK2*. Further analysis of the top 10 differentially regulated kinases at this time point revealed a significant transient increased expression of *PDK4*, *SQSTM1*, *BRAF*, *SIK1B*, *SIK2*, and *SIK3* (Figure 1C). Notably, none of these kinases have been studied in the context of pancreatic endocrinogenesis. All three SIK family members (*SIK1B*, *SIK2*, and *SIK3*) in the top 10 gene list suggest that SIKs may strongly link to endocrinogenesis. qPCR analysis validated the expression of *SIK1B*, *SIK2*, and *SIK3* (Figure 1D). Western blot analysis corroborated the *SIK3* mRNA data by demonstrating that SIK3 protein levels increased 3–6 h post LatB treatment (Figure 1E). These findings suggest that SIKs act downstream of F-actin dynamics during endocrinogenesis.

### Sodium chloride treatment promotes endocrinogenesis through increased SIK expression

**Figure 2:**
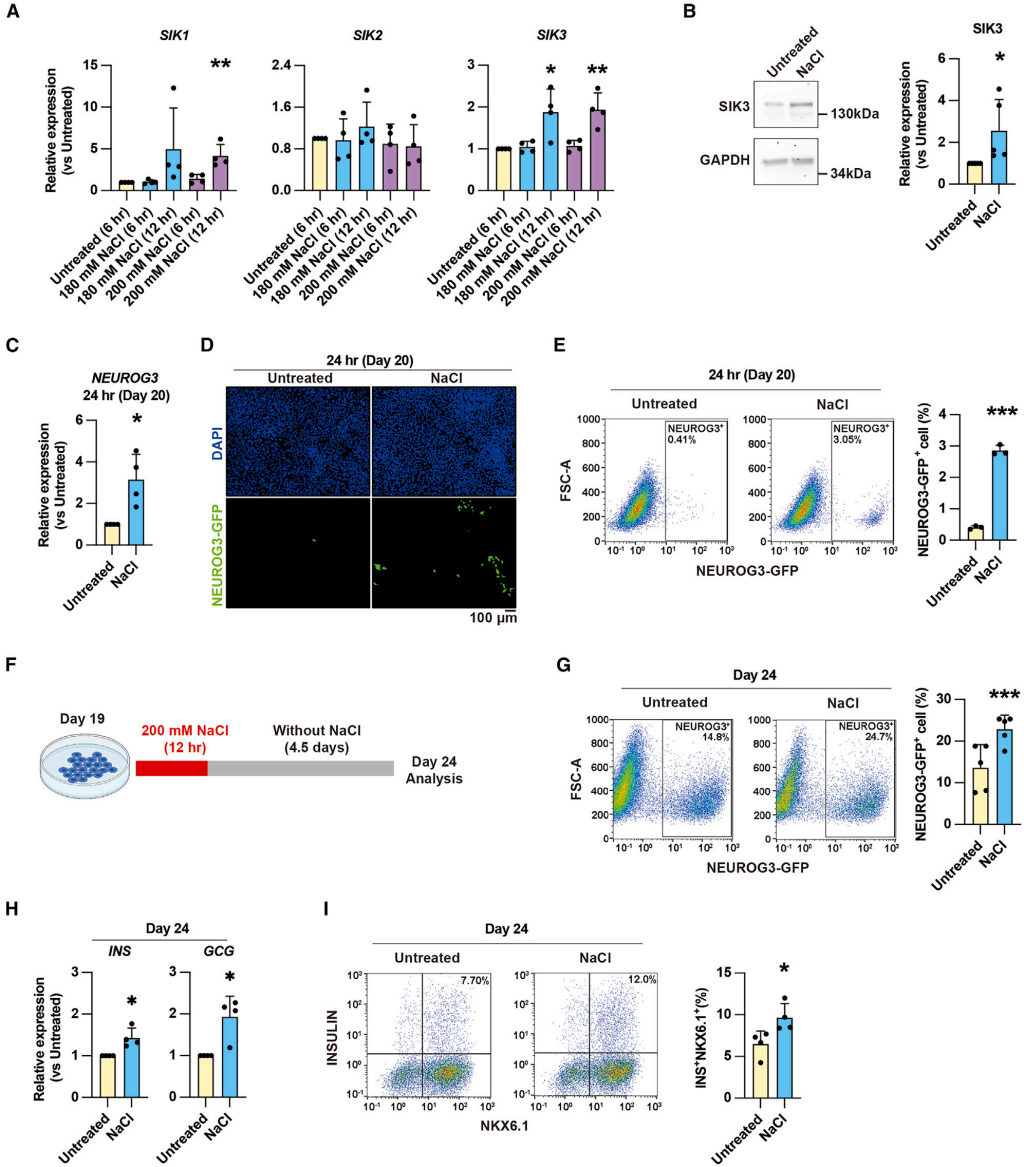
Sodium chloride promotes endocrine cell differentiation by increasing SIK expression (A) qPCR analysis of *SIK1*, *SIK2*, and *SIK3* expression after 180 and 200 mM sodium chloride (NaCl) treatment for 6 or 12 h. Data represent the mean ± SD (*n* = 4 independent repeats). The samples are derived from both the HuES4 and NEUROG3-GFP cell lines. (B) Western blot analysis of SIK3 protein levels after 200 mM NaCl treatment for 12 h. Data represent the mean ± SD (*n* = 5 independent repeats). The samples are derived from the NEUROG3-GFP cell line. (C) qPCR analysis of *NEUROG3* expression levels at 24 h after 200 mM NaCl treatment for 12 h. Data represent the mean ± SD (*n* = 4 independent repeats). The samples are derived from both the HuES4 and NEUROG3-GFP cell lines. (D) Imaging shows an increase in NEUROG3-GFP^+^ endocrine progenitors generated at 24 h after 12 h of 200 mM NaCl treatment compared to untreated cultures. Scale bar, 100 μm. (E) Flow cytometry-based quantification of NEUROG3-GFP^+^ cells at 24 h with and without 200 mM NaCl treatment for 12 h. Data represent the mean ± SD (*n* = 3 independent repeats). (F) Experimental setup for assessing endocrine cell differentiation after NaCl treatment: day 19 cells are treated with 200 mM NaCl for 12 h, then NaCl is removed, and cultures are maintained for 4.5 days (until day 24) for analysis. (G) Flow cytometry-based quantification of NEUROG3-GFP^+^ cells at day 24 with and without 200 mM NaCl treatment for 12 h. Data represent the mean ± SD (*n* = 5 independent repeats). (H) qPCR analysis of *INS* and *GCG* expression levels at day 24 with and without 200 mM NaCl treatment for 12 h. Data represent the mean ± SD (*n* = 4 independent repeats). The samples are derived from the NEUROG3-GFP cell line. (I) Flow cytometry-based quantification of INS^+^NKX6.1^+^ beta cells at day 24 with and without 200 mM NaCl treatment for 12 h. Data represent the mean ± SD (*n* = 4 independent repeats). The samples are derived from the NEUROG3-GFP cell line. ^∗^*p* < 0.05; ^∗∗^*p* < 0.01; ^∗∗∗^*p* < 0.001 (two-tailed paired Student’s t test). See also Tables S2 and S3.

SIKs can be exogenously activated by sodium homeostasis (Taub et al., 2010). Treatment with 180 mM sodium chloride (NaCl) for 12 h on day 19 promoted *SIK3* expression, while 200 mM NaCl treatment for the same duration promoted both *SIK1* and *SIK3* expression (Figure 2A). Western blot analysis confirmed that the SIK3 protein levels increased after 12 h with 200 mM NaCl treatment (Figure 2B). Based on these results, a 12-h treatment with 200 mM NaCl at day 19 was chosen to test whether increased SIK expression enhanced EP differentiation. 12-h treatment with 200 mM NaCl does not change the cell viability (Table S2). Consistent with the increased expression of *SIK1* and *SIK3*, *NEUROG3* expression also increased following NaCl treatment (Figure 2C). This observation was corroborated by immunofluorescence and flow cytometry, demonstrating an increased proportion and the number of NEUROG3-GFP^+^ EPs after NaCl treatment (Figures 2D and 2E; Table S3).

To further verify whether NaCl treatment promotes endocrine cell differentiation, NaCl-treated cells were cultured in a standard medium for an additional 4.5 days (until day 24) (Figure 2F). The NEUROG3-GFP reporter could be used to roughly estimate not only the number of EPs but also the total number of endocrine cells (Beydag-Tasoz et al., 2023; Lof-Ohlin et al., 2017). Flow cytometry revealed that the number of NEUROG3-GFP^+^ endocrine cells was significantly higher in the NaCl-treated cells compared to the untreated cells on day 24 of differentiation (Figure 2G). Consistently, the expression levels of *INS* and *GCG* were upregulated in the NaCl-treated cells compared to the untreated cells on day 24 (Figure 2H). Furthermore, NaCl treatment resulted in a 1.5-fold increase in beta cell number (Figure 2I). Collectively, these results demonstrate that NaCl treatment enhances pancreatic endocrine lineage specification and suggest that increased SIK expression is the underlying mechanism.

### SIKs promote endocrine cell differentiation

**Figure 3:**
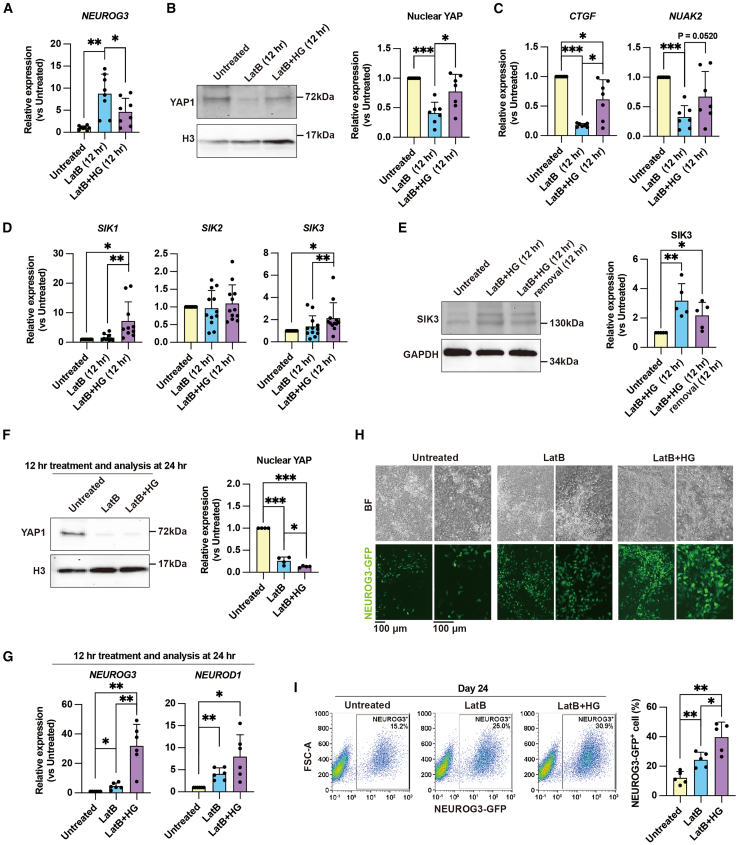
SIKs promote pancreatic endocrine cell differentiation by reducing nuclear YAP (A) qPCR analysis of *NEUROG3* expression levels at 12 h in untreated, LatB-treated, and LatB+HG-9-91-01 (HG)-treated cells. Data represent the mean ± SD (*n* = 8 independent repeats). The samples are derived from both the HuES4 and NEUROG3-GFP cell lines. (B) Western blot analysis of nuclear YAP1 at 12 h in untreated, LatB-treated, and LatB+HG-treated cells. H3 served as a loading control. Data represent the mean ± SD (*n* = 7 independent repeats). The samples are derived from the NEUROG3-GFP cell line. (C) qPCR analysis of *CTGF* and *NUAK2* (YAP target genes) expression levels at 12 h in untreated, LatB-treated, and LatB+HG-treated cells. Data represent the mean ± SD (*n* = 7 independent repeats). The samples are derived from both the HuES4 and NEUROG3-GFP cell lines. (D) qPCR analysis of *SIK1/2/3* expression levels at 12 h in untreated, LatB-treated, and LatB+HG-treated cultures. Data represent the mean ± SD (SIK1: *n* = 10; SIK2 and SIK3: *n* = 12, independent repeats). The samples are derived from both the HuES4 and NEUROG3-GFP cell lines. (E) Western blot analysis of SIK3 protein levels at 12 h in untreated and 12 h LatB+HG-treated cultures and 24 h in 12 h LatB+HG + 12 h LatB and HG removal cultures. Data represent the mean ± SD (*n* = 5 independent repeats). The samples are derived from the NEUROG3-GFP cell line. (F) Western blot analysis of nuclear YAP at 24 h in untreated, 12 h LatB-treated, and 12 h LatB+HG-treated cultures. H3 served as a loading control. Data represent the mean ± SD (*n* = 4 independent repeats). The samples are derived from the NEUROG3-GFP cell line. (G) qPCR analysis of *NEUROG3* and *NEUROD1* expression levels at 24 h in untreated, 12 h LatB-treated, and 12 h LatB+HG-treated cultures. Data represent the mean ± SD (*n* = 6 independent repeats). The samples are derived from NEUROG3-GFP cell lines. (H) Imaging shows an increase in NEUROG3-GFP^+^ endocrine progenitors generated on day 24 in 12 h LatB+HG-treated cultures compared to untreated or 12 h LatB cultures. Scale bar, 100 μm. (I) Flow cytometry-based quantification of NEUROG3-GFP^+^ cells on day 24 in untreated, 12 h LatB-treated, and 12 h LatB+HG-treated cultures. Data represent the mean ± SD (*n* = 5 independent repeats). ^∗^*p* < 0.05; ^∗∗^*p* < 0.01; ^∗∗∗^*p* < 0.001 (two-tailed paired Student’s t test). See also Figures S1 and S2; Tables S2 and S3.

In our previous work, we demonstrated that the mechanosensory YAP1 inhibits NEUROG3 expression by binding directly to its promoter (Mamidi et al., 2018). To assess whether SIKs promote endocrine cell differentiation via a reduction in nuclear YAP1, we pharmacologically inhibited SIK activity in LatB-treated cells. To account for potential redundancy among SIK family members, we used the pan-SIK inhibitor HG-9-91-01 (HG), which efficiently blocks the ATP-binding sites of all SIK isoforms (Clark et al., 2012). As expected, *NEUROG3* expression levels were lower in LatB+HG-treated cells compared to the cells treated with LatB alone (Figure 3A). This observation is most likely explained by restored nuclear levels of YAP1 upon HG treatment (Figure 3B). Consistently, the expression of YAP1 target genes *CTGF* and *NUAK2* increased following HG treatment (Figure 3C). These results suggest that the pro-endocrinogenic function of SIKs is mediated by reduced nuclear YAP1 levels. In light of the established link between reduced nuclear YAP1 and increased endocrinogenesis (Mamidi et al., 2018; Rosado-Olivieri et al., 2019), these results suggest that the pro-endocrinogenic function of SIKs involves the suppression of nuclear YAP1.

Interestingly, extended HG treatment has been reported to increase SIK transcription, possibly through a negative feedback mechanism (Wang et al., 2020). We confirmed this result, as HG treatment alone increased SIK expression (Figure S1C). Consistently, LatB+HG treatment for 12 h significantly increased the mRNA levels of *SIK1* and *SIK3* (Figure 3D). Given that increased SIK expression enhances endocrine cell differentiation, we hypothesized that removing HG after 12 h of treatment would lift the inhibition of SIK activity and enhance endocrine cell differentiation due to increased SIK expression. To test our hypothesis, LatB and HG were removed after 12 h, after which cells were cultured in a standard medium (hereon referred to as 12 h LatB+HG). Western blot analysis showed that the protein levels of SIK3 were significantly higher in LatB+HG-treated cells compared to untreated cells, and these elevated levels persisted for at least 12 h after LatB and HG removal (Figure 3E). As expected, nuclear YAP1 decreased in the 12 h LatB+HG cells compared to the untreated and LatB-treated cells (Figure 3F). Consistently, the expression of EP marker genes *NEUROG3* and *NEUROD1* was significantly higher in the 12 h LatB+HG cells compared to the untreated and LatB-treated cells (Figure 3G). As a result, the number of NEUROG3-GFP^+^ endocrine cells was significantly higher in the 12 h LatB+HG cells by day 24 of differentiation compared to the untreated and LatB-treated cells (Figure 3H; Tables S2 and S3). Consistent with the effects of 12 h LatB+HG treatment, HG treatment alone for 12 h also increases the expression of EP marker genes *NEUROG3* and *NEUROD1* at day 20, as well as the generation of NEUROG3-GFP^+^ endocrine cells at day 24 (Figures S1D–S1F; Table S3). Altogether, these data suggest that SIKs are required for endocrine differentiation.

To substantiate our *in vitro* findings *in vivo*, we treated E11.5 pancreatic explants with LatB+HG for 1 day, followed by LatB+HG removal and 2 days of culture (Figure S2A). The LatB+HG treatment significantly increased the expression levels of *Neurog3*, *Ins*, and *Gcg* compared to untreated controls and elevated *Neurog3* and *Gcg* expression levels compared to LatB treatment (Figure S2B). Consistently, whole-mount immunofluorescence analysis demonstrated that LatB+HG treatment significantly increased INS^+^ beta cell and GCG^+^ alpha cell generation (Figures S2C and S2D). The reduction in the Ecad area and associated reduced epithelial branching is likely a consequence of increased differentiation of bi-potent progenitors into NEUROG3^+^ cells following LatB+HG treatment (Figure S2B). This leads to fewer expandable bi-potent progenitors and a reduced number of Ecad^+^ progenitors. These NEUROG3^+^ cells subsequently differentiate into endocrine cells, such as GCG^+^ alpha cells and INS^+^ beta cells (Figures S2B and S2C). Similar observations have been made in other mouse models where endocrinogenesis was enhanced, such as in *Yap1* or *Hes1* knockout mice (Jensen et al., 2000; Mamidi et al., 2018). In summary, our findings indicate that increased SIK1 and SIK3 expression promotes endocrinogenesis by reduced levels of nuclear YAP1.

### Increased SIK expression promotes the generation of functional beta cells

**Figure 4:**
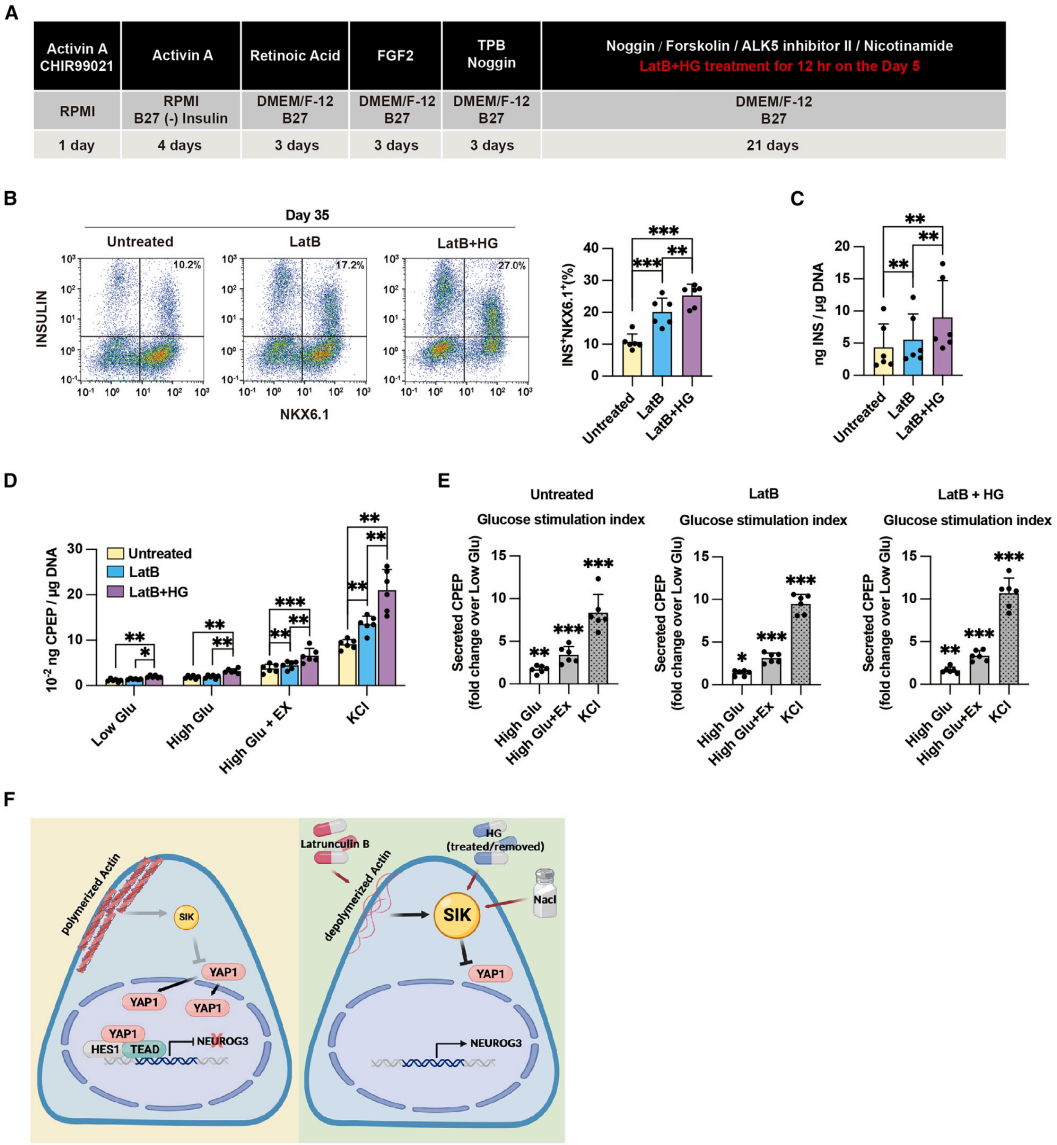
HG promotes functional beta cell generation (A) Modified protocol for differentiation of hESCs into pancreatic beta cells. At the last stage of differentiation, LatB and HG are added on day 19 of the whole differentiation for 12 h. The cells are collected on day 35 for analysis. (B) Flow cytometry-based quantification of INS^+^NKX6.1^+^ beta cells at day 35 in untreated, 12 h LatB-treated, and 12 h LatB+HG-treated cultures. Data represent the mean ± SD (*n* = 6 independent repeats). (C) ELISA measurements of total INS content normalized to DNA content in day 35 cells. Data represent the mean ± SD (*n* = 6 independent repeats). (D) ELISA measurements of secreted C-peptide after 30 min in 1.67 mM glucose (low Glu), 16.67 mM glucose (high Glu), 16.67 mM glucose plus 10 nM exendin-4 (high Glu+Ex), and 1.67 mM glucose with 30 mM KCl (KCl) condition. The C-peptide secretion normalized to DNA content in day 35 cells. Data represent the mean ± SD (*n* = 6 independent repeats). (E) The fold change of C-peptide secretion in 30 min high Glu, high Glu+Ex, and KCl relative to low Glu. Asterisks indicate the significance between each condition and low Glu. Data represent the mean ± SD (*n* = 6 independent repeats). (F) Model illustrating the proposed mechanism for SIKs transducing F-actin-triggered bi-PP fate decisions. Actin depolymerization increases SIK expression, which reduces nuclear YAP1 levels and increases NEUROG3 expression. Pharmacological induction of SIK expression, through treatments with LatB, NaCl, or HG-9-91-01 (HG), promotes NEUROG3 expression and endocrinogenesis. ^∗^*p* < 0.05; ^∗∗^*p* < 0.01; ^∗∗∗^*p* < 0.001 (two-tailed paired Student’s t test). The samples are derived from both SA121 (*n* = 4 independent repeats) and HuES4 (*n* = 2 independent repeats) cell lines. See also Figure S3; Table S4.

To investigate whether EPs derived from LatB+HG treatment can further differentiate into functional beta cells, we extended the current differentiation protocol until day 35 (Figure 4A). At day 35, LatB+HG-treated cultures contained more beta cells (25.35% ± 3.43%) compared to untreated (10.75% ± 2.48%) and LatB-treated (20.10% ± 4.37%) cultures (Figures 4A and 4B). As expected, the cells expressed characteristic beta cell markers, including C-peptide, NKX6.1, GCG, PDX1, and NEUROD1 (Figure S3). Additionally, due to an increased number of beta cells in the cultures, the combined treatment of LatB and HG shows more insulin content in the cultures than that in untreated and LatB-treated cultures (Figure 4C). Furthermore, LatB+HG treatment resulted in the formation of beta cells that secreted C-peptide in response to high glucose and exendin-4 (Figures 4D and 4E; Table S4). However, the absence of a significant difference in the glucose stimulation index among the untreated, LatB, and LatB+HG groups suggests that LatB and HG treatments do not improve beta cell maturation (Table S4). These findings suggest that increased SIK expression following LatB+HG treatment promotes the differentiation of glucose-responsive beta cells.

## Discussion

Previous studies demonstrated that changes in ECM interactions control bi-PP fate via the F-actin-YAP1-Notch mechanosignaling axis (Mamidi et al., 2018). However, the exact mechanism by which F-actin dynamics regulate nuclear YAP1 levels remains unclear. Here, we identify SIKs as pivotal downstream mediators of F-actin dynamics in the specification of bi-PPs into EPs. Actin depolymerization induced by LatB increases SIK expression, leading to reduced nuclear YAP1 levels and enhanced NEUROG3 expression (Figure 4F). Notably, pharmacological induction of SIK expression promotes endocrinogenesis and the generation of functional glucose-responsive beta cells.

YAP is a key effector in mechanotransduction (Dupont et al., 2011), governing gene expression changes driven by mechanical signals that shape cell fate decisions during the development of organs, such as the pancreas (Mamidi et al., 2018; Rosado-Olivieri et al., 2019), trophectoderm (Nishioka et al., 2009), kidney (Reginensi et al., 2013), and lung (Mahoney et al., 2014). Furthermore, the link between SIKs and YAP appears to be a widespread phenomenon. For instance, SIK2 and SIK3 regulate the Hippo/YAP pathway in *Drosophila* (Wehr et al., 2013), and SIK1 decreases nuclear YAP levels in mouse and human smooth muscle cells (Pu et al., 2022). These findings suggest that SIKs are conserved regulators of YAP and may play a broader role than previously anticipated in organogenesis and cell fate specification.

SIK expression and activity are influenced by various processes. Here, we demonstrate that mechanical tension, mediated through F-actin dynamics, affects SIK expression. However, the underlying mechanism for this observation remains unclear. One possible explanation is that F-actin may regulate SIK expression by modulating intracellular sodium homeostasis through its effect on ion channels. This hypothesis is supported by evidence that reduction of F-actin levels can increase intracellular Na^+^ concentrations via the activation of epithelial Na^+^ channels in polarized epithelial cells of the kidney, lung, and colon (Kleyman and Eaton, 2020; Morachevskaya and Sudarikova, 2021). Additionally, SIK activity can be regulated independently of F-actin. For instance, Ca^2+^ modulates intracellular sodium levels through the Na^+^/Ca^2+^ exchanger (NCE1) and calcium/calmodulin-dependent protein kinase, increasing or decreasing SIK activity (Sjostrom et al., 2007). Moreover, metabolic stress-induced activation of liver kinase B1 can phosphorylate and activate SIKs (Shackelford and Shaw, 2009). These findings indicate that regulation of metabolic changes or intracellular Na^+^ and Ca^2+^ levels in bi-PPs may also enhance endocrinogenesis via increased SIK expression and/or activity.

Bioreactor-based 3D differentiation of hPSCs is currently the most favorable approach for the scalable engineering of pancreatic beta cells. However, directly controlling endocrinogenesis by manipulating the ECM within a 3D system poses challenges. Additionally, the use of actin-depolymerizing drugs, such as LatB, may lead to significant morphological changes or even disaggregation of the cell clusters. Our findings offer alternative strategies for regulating endocrinogenesis that do not rely on ECM components or actin-depolymerizing agents. Here, we show that combined treatment with NaCl and HG boosts endocrinogenesis and beta cell differentiation via enhanced SIK activity, making them promising candidates for similar applications under scalable conditions.

In summary, our findings provide valuable new insights into the molecular mechanisms underlying pancreatic endocrine differentiation, emphasizing a crucial role of SIKs as intermediates in the F-actin-YAP1 signaling axis. Additionally, this study introduces SIKs as new targets for boosting beta cell generation from hPSCs.

## Methods

### Quantification and statistical analysis

Statistical analyses were performed with GraphPad Prism (version 7.0 or 8.0, GraphPad Software). Unless otherwise noted, a paired nonparametric test (Wilcoxon matched-pairs signed-rank test) was used to assess significance. An unpaired nonparametric test was used for unpaired data (Mann-Whitney test). Asterisks denote *p* values as follows: ^∗^*p* < 0.05; ^∗∗^*p* < 0.01; ^∗∗∗^*p* < 0.001. Each N represents a biological replicate (mice fetal pancreas or independent experiments). Data figures illustrate the mean with standard deviation (SD) or with standard error of the mean (SEM) and the values of individual biological replicates.

## Resource availability

### Lead contact

Further information and requests for resources and reagents should be directed to and will be fulfilled by the lead contact, Henrik Semb (henrik.semb@helmholtz-munich.de).

### Materials availability

All unique/stable reagents in this study are available from the lead contact with a completed Materials Transfer Agreement.

### Data and code availability

• The accession number for the RNA-seq datasets reported in this paper is GSE275775. They are publicly available as of the date of publication.
• This paper does not report original code.
• Any additional information required to reanalyze the data reported in this paper is available from the lead contact upon request.

## Acknowledgments

We thank Anna Øster for her excellent technical support, Anna Månsson for helping with animal experiments, and Ivan Kulik for assistance with the immunofluorescence. All the work is supported by the European Union’s Horizon 2020 research and innovation program (ISLET, no. 874839), the Novo Nordisk Foundation Center for Stem Cell Biology (DanStem) at the 10.13039/501100001734University of Copenhagen (10.13039/501100009708NNF grant, NNF17CC0027852), and the 10.13039/501100013295Helmholtz Zentrum München.

## Author contributions

C.T., A.R., C.E., A.M., and H.S. designed and interpreted the experiments. C.T. performed most of the experiments and analyses. A.R. performed and analyzed RNA-seq data. C.E. performed and analyzed fetal mouse pancreas experiments. A.M. performed some of the *in vitro* differentiation and data analysis. C.T. and H.S. wrote the manuscript.

## Declaration of interests

H.S. is a member of the Stem Cell Reports editorial board.

## Supplemental information

Document S1. Figures S1–S4 and Tables S2–S4

Table S1. Summary of differentially expressed genes, KEGG, and the Gene Ontology analysis at each time point, related to Figure 1

Document S2. Article plus supplemental information
