# Supplementary material for: Salt-inducible kinases transduce mechanical forces into the specification of the pancreatic endocrine lineage
Source: Stem Cell Reports. 2025 Mar 6;20(4):102444. doi: 10.1016/j.stemcr.2025.102444 (PMC12069894; doi:10.1016/j.stemcr.2025.102444)
Supplement: Document S1. Figures S1–S4 and Tables S2–S4 [file mmc1.pdf]

Stem Cell Reports, Volume *20*

Supplemental Information

Salt-inducible kinases transduce mechanical forces into the specification of the pancreatic endocrine lineage

Chenglei Tian, Adam Rump, Christine Ebeid, Anant Mamidi, and Henrik Semb

Supplemental Information

Salt-inducible kinases transduce mechanical forces into the specification of the pancreatic endocrine lineage

Chenglei Tian, Adam Rump, Christine Ebeid, Anant Mamidi, Henrik Semb

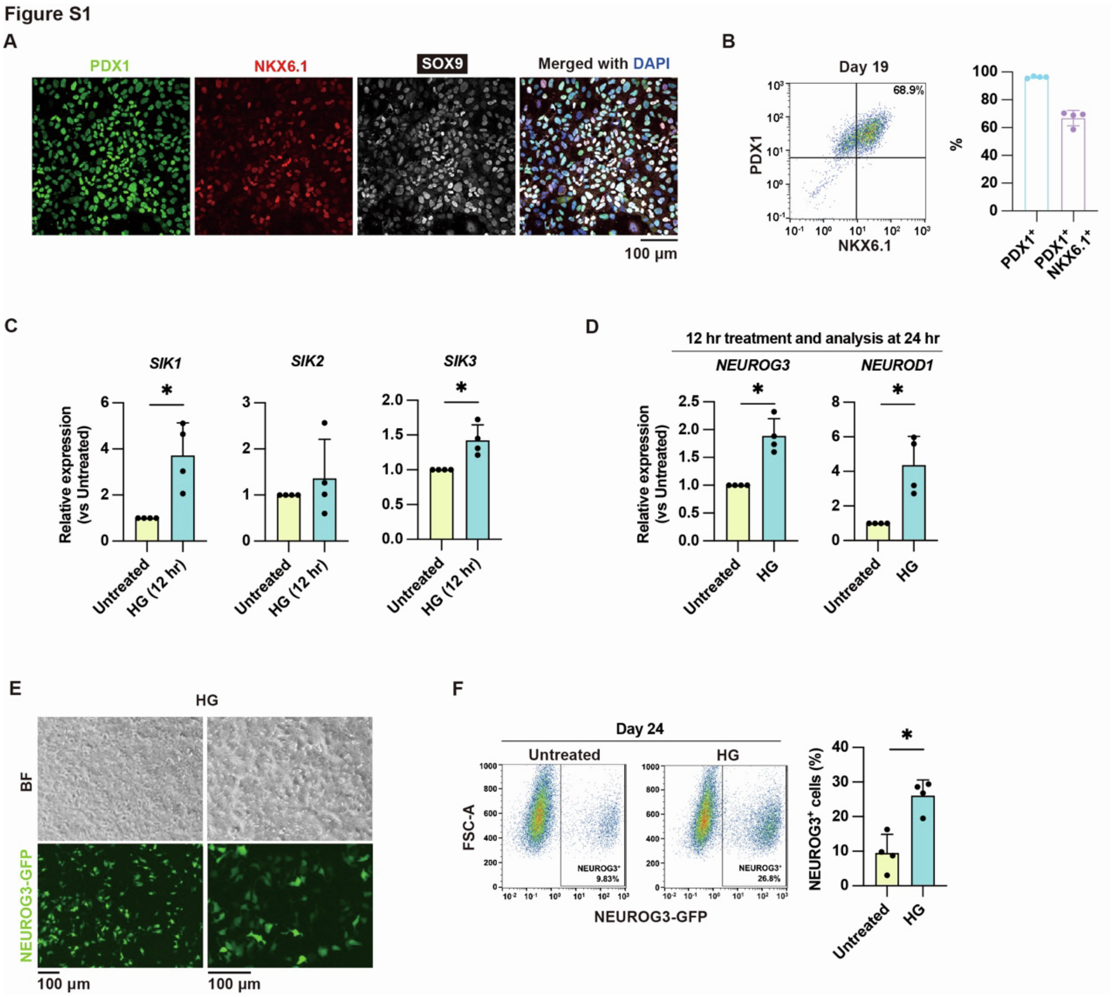

Supplementary Figure 1. Identification of bi-potent pancreatic progenitors (bi-PPs) at Day 19 and HG increases *SIK1* and *SIK3* expression, related to Figures 1 and 3.

(A) Typical confocal images of PDX1, NKX6.1, and SOX9 expression in D19 hESC-derived bi-PPs. Scale bar, 100 μm. The samples are derived from the HuES4 cell line.

(B) Flow cytometry-based quantification of PDX^{+}NKX6.1^{+} in D19 hESC-derived bi-PPs. Data represent the mean ± SD (n = 4 independent repeats). The samples are derived from the HuES4 cell line.

(C) qPCR analysis of *SIK1*, *SIK2*, and *SIK3* expression levels at 12 hr in untreated and HG-treated cultures. Data represent the mean ± SD (n = 4 independent repeats). The samples are derived from the NEUROG3-GFP cell line.

(D) qPCR analysis of *NEUROG3* and *NEUROD1* expression levels at 24 hr in untreated and 12 hr HG-treated cultures. Data represent the mean ± SD (n = 4 independent repeats). The samples are derived from NEUROG3-GFP cell lines.

(E) Imaging shows NEUROG3-GFP^{+} endocrine progenitors generated on Day 24 in 12 hr HG-treated cultures. Scale bar, 100 μm.

(F) Flow cytometry-based quantification of NEUROG3-GFP^{+} cells on Day 24 in untreated and 12 hr HG-treated cultures. Data represent the mean ± SD (n = 4 independent repeats).

*, $p<0.05$ (two-tailed paired Student's t-test).

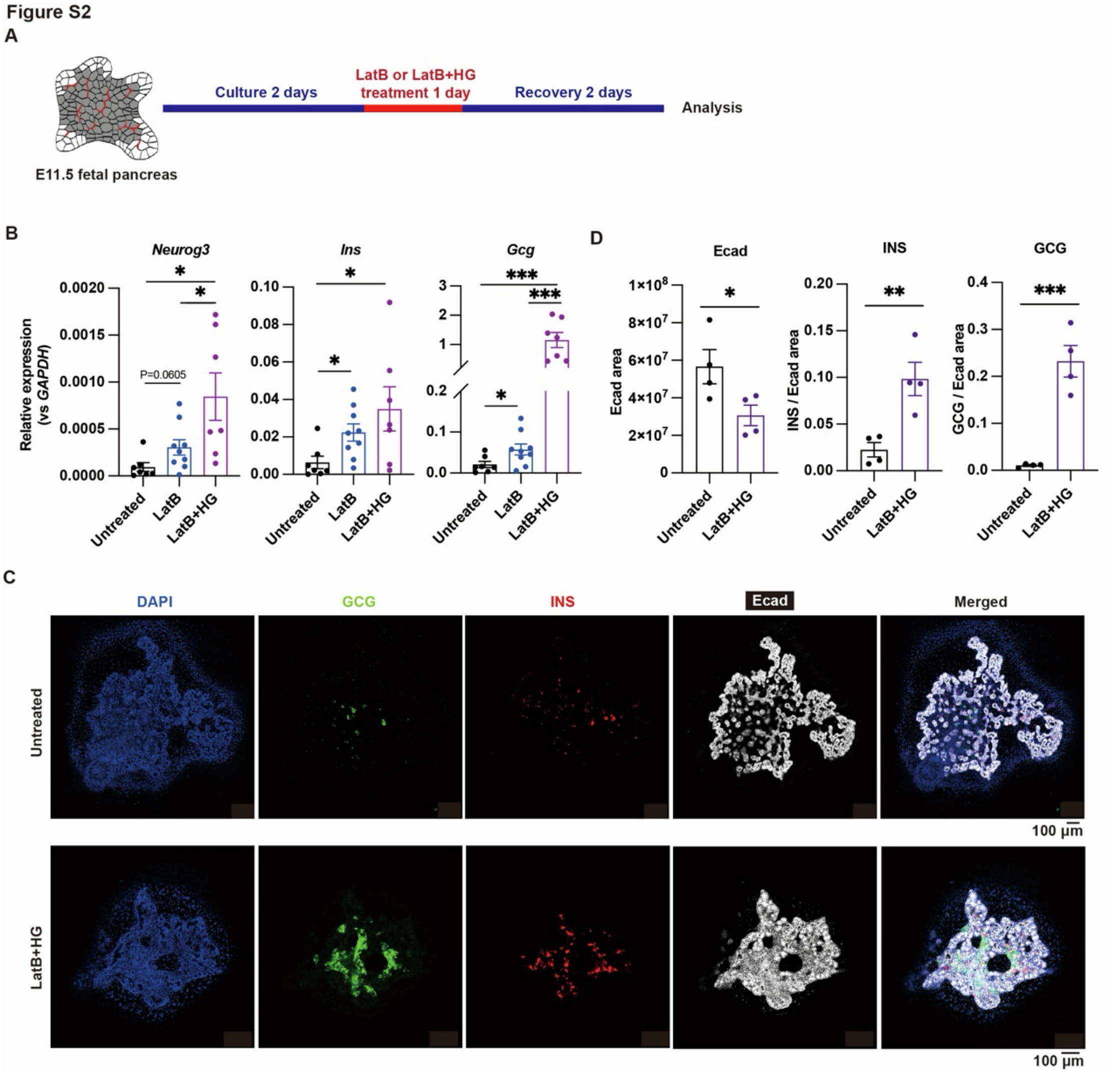

Supplementary Figure 2. LatB+HG promotes endocrine cell differentiation in *ex vivo* mouse embryonic pancreas, related to Figure 3.

(A) Experimental setup for assessing endocrine cell differentiation after LatB+HG treatment in mouse embryonic pancreas: E11.5 pancreata are cultured for 2 days, treated with LatB or LatB+HG for 1 day, and then cultured for an additional 2 days without treatment before analysis.

(B) qPCR analysis of *Neurog3*, *Ins*, and *Gcg* expression in untreated, LatB treated, and LatB+HG-treated fetal pancreas. Data represent the mean ± SEM (Untreated and LatB+HG: n = 7 fetal pancreas; LatB: n = 9 fetal pancreas).

(C) Typical whole-mount immunostaining of GCG, INS, and Ecad expression in *ex vivo* cultured embryonic pancreas. Scale bar, 100 μm.

(D) Image-based quantification of E-cadherin $(Ecad)^+$, INS^+ or GCG^+ area in untreated and LatB+HG-treated *ex vivo* embryonic pancreas. Ecad, which marks all epithelial areas, is used for normalization. Data represent the mean ± SD (n = 4 fetal pancreas).

*, $p<0.05$; **, $p<0.01$; ***, $p<0.001$ (the unpaired two-samples Wilcoxon test).

Figure S3

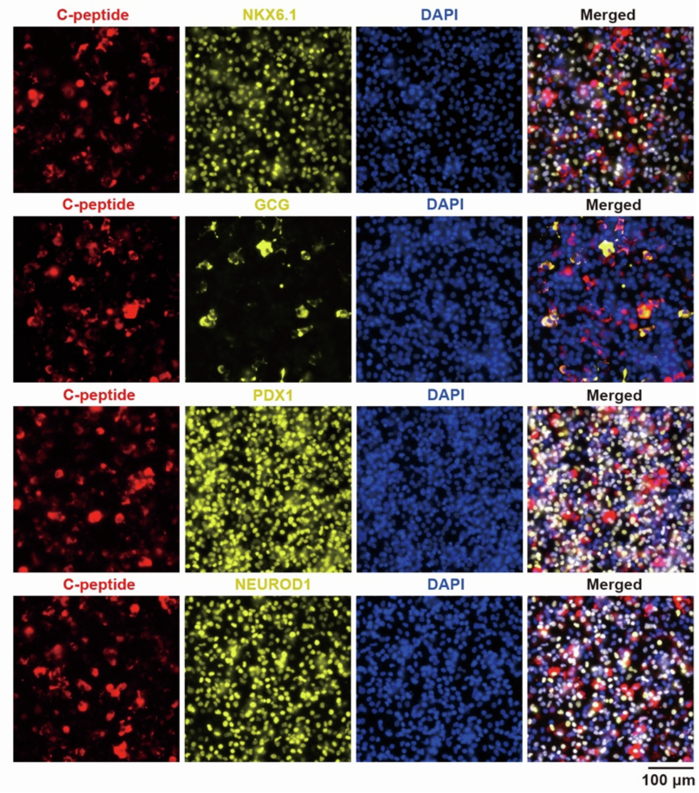

Supplementary Figure 3. Immunofluorescence analysis of C-peptide, NKX6.1, GCG, PDX1, and NEUROD1 expression in LatB+HG-treated D35 cultures, related to Figure 4. Scale bar, 100 µm. The samples are derived from the SA121 cell line.

Figure S4

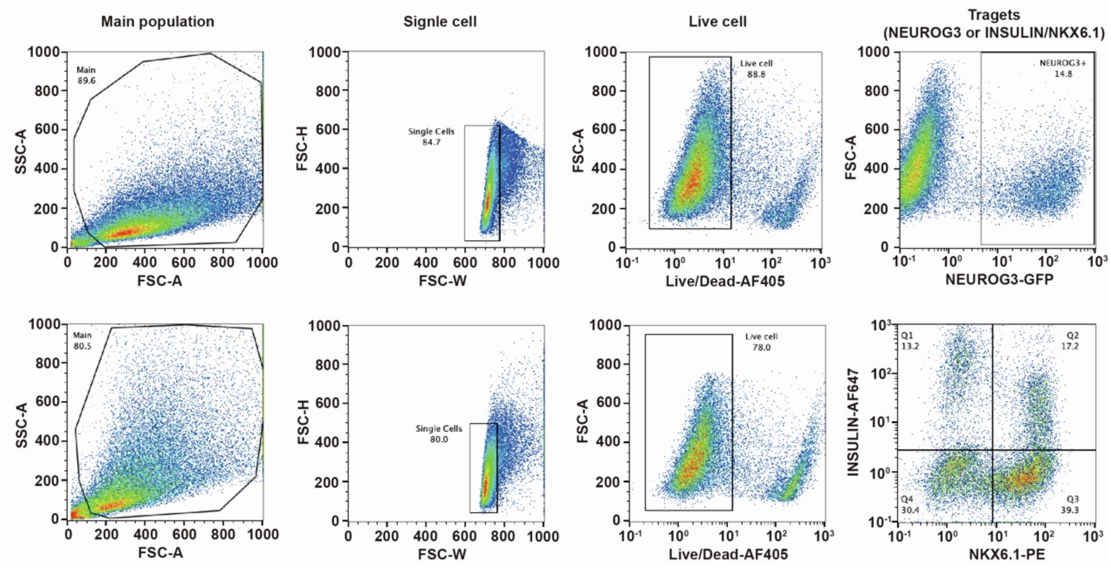

Supplementary Figure 4. Examples of FACS gating strategy, related to Figures 2, 3, and 4.

Supplementary Table 1. Summary of differentially expressed genes, KEGG, and the Gene Ontology analysis at each time point, related to Figure 1.

Supplementary Table 2. Summary of the cell viability by Live/Dead staining after 12 hr treatment, related to Figures 2 and 3.

	Untreated	200 mM NaCl	LatB	HG	LatB + HG
Viability (%)	88.58±7.97	86.85±8.85	76.60±13.01	83.45±9.63	72.83±11.27*

Asterisks indicate the significance between each condition and Untreated. Data represent the mean ± SD (n = 4 independent repeats).

*, p<0.05 (two-tailed paired Student's t-test).

Supplementary Table 3. Summary of total cell and NEUROG3-GFP$^+$ cell numbers on Day 24, related to Figures 2, 3 and 4.

	Untreated	200 mM NaCl	LatB	HG	LatB + HG
Total cell number (10^6/12-well)	1.40±0.12	1.09±0.20	1.06±0.27*	0.96±0.24	0.67±0.10**
NEUROG3-GFP$^+$ cell number (10^6/12-well)	0.13±0.07	0.18±0.06	0.25±0.05	0.24±0.05*	0.27±0.05*

Asterisks indicate the significance between each condition and Untreated. Data represent the mean ± SD (n = 4 independent repeats).

*, p<0.05; **, p<0.01 (two-tailed paired Student's t-test).

Supplementary Table 4. Summary of the fold change of C-peptide secretion in 30 minutes High Glu, High Glu+Ex, and KCl relative to Low Glu in all the groups, related to Figure 4.

	Untreated	LatB	LatB + HG
High Glu	1.71±0.43**	1.37±0.32*	1.65±0.36**
High Glu + Ex	3.41±0.98***	3.13±0.58***	3.37±0.61***
KCl	8.35±2.14***	9.49±1.10***	10.69±1.76***

Asterisks indicate the significance between each condition and Low Glu. Data represent the mean ± SD (SA121 cell line: n = 4 and HuES4 cell line: n = 2, independent repeats).

*, p<0.05; **, p<0.01; ***, p<0.001 (two-tailed paired Student's t-test).

Supplemental Experimental Procedures

KEY RESOURCES TABLE

REAGENT or RESOURCE	SOURCE	IDENTIFIER
**Antibodies**		
Alexa fluor 647 anti-C-peptide	BD Pharmingen	# 565831<br>RRID: AB_2739371
Alexa fluor 647 anti-INSULIN	BD Pharmingen	#565689<br>RRID: AB_2739331
PE anti-NKX6.1	BD Pharmingen	#563023<br>RRID: AB_2716792
GLUCAGON	Cell Signaling	#8233<br>RRID: AB_10859908
PDX1	R&D Systems	#AF2419<br>RRID: AB_355257
NKX6.1	DSHB	#F55A12<br>RRID: AB_532379
SOX9	Sigma Aldrich	#HPA001758<br>RRID: AB_1080067
NEUROD1	Proteintech	#12081-1-AP<br>RRID: AB_2877823
GAPDH	R&D Systems	#AF5718<br>RRID: AB_2278695
H3	Abcam	#ab1791<br>RRID: AB_302613
YAP1	Cell Signaling	#14074<br>RRID: AB_2650491
SIK3	Abcam	#ab88495<br>RRID: AB_2042747
SIK3	Cell Signaling	#39477<br>RRID: AB_3251492
**Chemicals, peptides, and recombinant proteins**		
CHIR99021	Axon Medchem	#1386
Activin A	PeproTech	#120-14
FGF2	PeproTech	#100-18B
Retinoic acid	Sigma Aldrich	#R2625
TBP	Calbiochem	#565740
ALK5iII	Santa Cruz	#sc-221234A
Noggin	PeproTech	#120-10C
Nicotinamide	Sigma Aldrich	#481907
Forskolin	Sigma Aldrich	#F6886
HG-9-91-01	Selleckchem	#S8393
Latrunculin B	Sigma Aldrich	#L5288
Bovine serum albumin (BSA)	Sigma Aldrich	#B4287
Y-27632	Merck Millipore	#688000
Biolaminin 521 LN (LN521)	Bio Lamina	#LN521-05
B27 supplement minus Insulin	Thermo Fisher	#A1895601
B27 supplement	Thermo Fisher	#17504001
Sodium Chloride (NaCl)	Sigma Aldrich	#S3014
Exendin-4	Sigma Aldrich	#E7144
**Critical commercial assays**		

RNeasy Mini Kit	Qiagen	#74106
iScript cDNA Synthesis Kit	BIO-RAD	#1708891
Subcellular Protein Fractionation Kits	Thermo Fisher	#78840
LIVE/DEAD™ Fixable Violet Dead Cell Stain Kit	Thermo Fisher	#L34964
Ultrasensitive C-peptide ELISA	Mercodia	#10-1141-01
Insulin ELISA	Mercodia	#10-1113-10
**Deposited data**		
RNA-seq datasets	This paper	GSE275775
**Experimental models: Cell lines**		
SA121	Takara bio	BioSamples: SAMEA6407434
NEUROG3-GFP (SA121)	Zarah M. Löf-Öhlin, et al (Lof-Ohlin et al., 2017)	NA
HuES4	Harvard University	BioSamples: SAMEA114214019
**TaqMan™ probes**		
GAPDH	Thermo Fisher	Hs02758991
HPRT1	Thermo Fisher	Hs01102345
RPL37A	Thermo Fisher	Hs99999909
SIK1	Thermo Fisher	Hs02573456
SIK2	Thermo Fisher	Hs01568566
SIK3	Thermo Fisher	Hs00228549
NEUROG3	Thermo Fisher	Hs01875204
NEUROD1	Thermo Fisher	Hs00159598
INS	Thermo Fisher	Hs02741908
GCG	Thermo Fisher	Hs01031536
Gapdh	Thermo Fisher	Mm99999915
Neurog3	Thermo Fisher	Mm00437606
Ins	Thermo Fisher	Mm01950294
Gcg	Thermo Fisher	Mm00801714
**Experimental models: Organisms/strains**		
C57BL/6 wildtype mice	In house	N/A
**Software and algorithms**		
Adobe Photoshop 2022	Adobe	https://www.adobe.com
Adobe Illustrator 2023	Adobe	https://www.adobe.com
Fiji 2.0/ImageJ	NIH Image	http://imagej.nih.gov/ij
GraphPad Prism 10	GraphPad	https://www.graphpad.com
FlowJo 10	BD	https://www.flowjo.com
RStudio	RStudio	https://posit.co/downloads/

The use of human embryonic stem cell (hESC) lines

SA121 (XY, RRID: CVCL_B296), NEUROG3-GFP (generated based on SA121 hESC line), and HuES4 (XY, RRID: CVCL_B172) hESC lines were used in this paper. SA121 cell line is from Takara Bio (Y00020), and the HuES4 line is from Harvard University. The karyotype of

hESCs was normal. All hESC lines were cultured on Biolaminin 521 LN (LN521) according to the manufacturer's protocol with daily changes of NutriStem® hPSC XF Medium (Sartorius, # 05-100-1A) at 37 °C and 5% CO_2. Cells were passaged twice to three times (seeding as 40,000 -60,000 cells/cm^2) weekly at subconfluency using StemPro™ Accutase™ (Thermo Fisher, #A1110501). The medium was supplemented with 10 µM ROCK inhibitor (Y-27632) on the first day post-passaging. hESCs were frozen by CryoStor cryopreservation media (Sigma, #C2874). hESCs were sterile and tested negative for mycoplasma in regular tests. All experiments were conducted using cells within 2 to 10 passages after thawing.

The use of animals

Mice were housed at the University of Copenhagen, and all experiments were performed according to guidelines and ethics approved by the Danish Animal Experiments Inspectorate (Dyreforsøgstilsynet). Data were collected from both male and female embryos.

hESC differentiation

hESCs were re-seeded on LN521 for pancreatic differentiation at 80,000 cells/cm^2. At 100% confluency, cells were induced to be further differentiated into pancreatic lineages following the Ameri et al. protocol (Ameri et al., 2017). For Latrunculin B (LatB) treatment, 0.5 µM LatB was added to the protocol for up to 24 hours on Day 19 to enhance endocrine differentiation (Mamidi et al., 2018). For NaCl treatment, since DMEM/F12 medium (Thermo Fisher, #11320033) contains ~120 mM NaCl, 60 mM (180 mM NaCl totally in the medium) or 80 mM (200 mM NaCl totally in the medium) NaCl was added to the protocol for 6 or 12 hours on Day 19. For LatB+HG-9-91-01 (HG) treatment, 4 µM HG was added alone or plus 0.5 µM LatB to the protocol for 12 hours on Day 19.

Ex vivo culture of mouse embryonic pancreas

The dorsal pancreata of E11.5 mouse embryos were micro-dissected and cultured on fibronectin-coated ibidi µ-Slide 8 well (Percival and Slack, 1999). Slides are precoated for 45 minutes at room temperature with 0.1 µg/µl fibronectin (Thermo Fisher, #33010-018). The explant culture media comprised M-199 Media plus phenol red (Sigma Aldrich, #M2154), supplemented with 10% fetal bovine serum (Thermo Fisher, #16140071), 1% Penicillin/Streptomycin (Thermo Fisher, #15140122), and 0.5% Fungizone (Sigma Aldrich, #A2411). The media was changed every other day. Explants were cultured at 37°C for up to 5 days. For treatment, 0.5 µM LatB and 4 µM HG were added to the medium for 1 day after 2 days of *ex vivo* culture, followed by removal of the treatment. Pancreata were left to recover for 2 days.

Flow cytometry analysis

Cells were dissociated by StemPro™ Accutase™. The LIVE/DEAD™ Fixable Blue Dead Cell Stain Kit (Thermo Fisher, #L23105) distinguishes live and dead cells. Then, cells were fixed and stained as previously described (Mamidi *et al.*, 2018) on a BD LSR Fortessa or Miltenyi MACSQuant analyzer. The gating strategies are shown in Figure S4. Antibodies are listed in the Key Resource Table.

Immunofluorescence staining

Fixation and immunological staining of hESC differentiated cells were performed as previously described(Mamidi *et al.*, 2018). Cells were fixed in 3.7% formalin for 20 minutes, permeabilized by 0.5% Triton X-100 for 15 minutes and blocked with 5% donkey serum for 1 hour. Primary antibodies are listed in the Key Resource Table. All Alexa Fluor-conjugated secondary antibodies (Thermo Fisher) were used as 1: 500 dilutions. Samples were imaged with Zeiss LSM780 confocal microscopes or the X-light V3 (CrestOptics) spinning disk with the Hamamatsu ORCA-flash camera. Image analyses were performed with Fiji (ImageJ).

RT-qPCR

Total RNA was extracted using the RNeasy Mini Kit (Qiagen, Cat#74106). According to the manufacturer's instructions, reverse transcription was performed with iScript cDNA Synthesis Kit (BIO-RAD, Cat#1708891). Real-time PCR measurements were performed in technical duplicates using the QuantStudio 7 Flex Real-Time-PCR-System (Thermo Fisher) with TaqMan FAM probes (Thermo Fisher) and TaqMan Master Mix (Thermo Fisher, Cat#4364103). Relative gene expression was determined using the housekeeping genes *GAPDH*, *HPRT1* or *RPL37A*. TaqMan probes and primers are listed in the Key Resource Table.

Western blot

Cells were washed twice in PBS, collected, and lysed in RIPA buffer (Thermo Fisher, Cat#8990) on ice for 30 min and then sonicated for 1 min at 5 s intervals. After centrifugation at 10,000 *g* at 4 °C for 10 min, the supernatant was transferred into new tubes. The concentration of the cell protein sample was measured by bicinchoninic acid, and protein samples were boiled in an SDS sample buffer at 95 °C for 5 min. The Subcellular Protein Fractionation Kit was used for nuclear protein isolation. The cell extract sample was resolved by 8-20% Acr-Bis SDS-PAGE and transferred to polyvinylidene difluoride membranes (PVDF, Merck Millipore). Nonspecific binding was blocked by incubation in 3% non-fat milk in TBS at room temperature for 1 h. Blots were then probed with primary antibodies overnight by incubation at 4 °C. GAPDH or H3 served as a loading control. Immunoreactivity bands were then probed for 1 h at room temperature with horseradish peroxidase (HRP)-conjugated secondary antibodies. Protein

bands were detected by SuperSignal™ Western Blot substrate (Thermo Fisher, Cat#A45915) or Chemiluminescent HRP substrate (Thermo Fisher, Cat#34579). Protein bands were quantified by densitometry using Fiji (ImageJ) software.

Glucose-stimulated insulin secretion (GSIS) assays

At Day 30, cells were dissociated for 5 min with StemPro™ Accutase™, reseeded on 24-well dishes at a density of 500,000 per cm^2, and cultured in final stage media for 5 days. On the day of the GSIS assays, cells were washed twice with Krebs-Ringer bicarbonate buffer (KRB) containing 115 mM NaCl, 5 mM KCl, 1 mM $MgCl_2$, 2.2 mM $CaCl_2$, 20 mM HEPES, 24 mM $NaHCO_3$ and 0.2 % BSA, and pH adjusted to 7.4. All subsequent incubation steps were carried out at 37 °C and 5% CO_2. For GSIS assays, the cells were pre-incubated for 1.5 hrs in KRB supplemented with 1.67 mM glucose before consecutive 30 min incubations in the first KRB with low glucose (1.67 mM), high glucose (16.67 mM), high glucose plus with 10 nM Exendin4 (Ex4) and finally low glucose with KCl (30 mM total). After each step, the medium was collected, and the cells were washed once with KRB. All samples were stored at -80 °C until analysis in technical duplicates with commercially available ELISA kits for INSULIN content and secreted human CPEP according to the manufacturer's recommendations.

Reference

Ameri, J., Borup, R., Prawiro, C., Ramond, C., Schachter, K.A., Scharfmann, R., and Semb, H. (2017). Efficient Generation of Glucose-Responsive Beta Cells from Isolated GP2(+) Human Pancreatic Progenitors. Cell Rep *19*, 36-49. 10.1016/j.celrep.2017.03.032.

Lof-Ohlin, Z.M., Nyeng, P., Bechard, M.E., Hess, K., Bankaitis, E., Greiner, T.U., Ameri, J., Wright, C.V., and Semb, H. (2017). EGFR signalling controls cellular fate and pancreatic organogenesis by regulating apicobasal polarity. Nat Cell Biol *19*, 1313-1325. 10.1038/ncb3628.

Mamidi, A., Prawiro, C., Seymour, P.A., de Lichtenberg, K.H., Jackson, A., Serup, P., and Semb, H. (2018). Mechanosignalling via integrins directs fate decisions of pancreatic progenitors. Nature *564*, 114-118. 10.1038/s41586-018-0762-2.

Percival, A.C., and Slack, J.M. (1999). Analysis of pancreatic development using a cell lineage label. Exp Cell Res *247*, 123-132. 10.1006/excr.1998.4322.
