# Supplementary material for: Salt-inducible kinases transduce mechanical forces into the specification of the pancreatic endocrine lineage
Source: Stem Cell Reports. 2025 Mar 6;20(4):102444. doi: 10.1016/j.stemcr.2025.102444 (PMC12069894; doi:10.1016/j.stemcr.2025.102444)
Supplement: Document S2. Article plus supplemental information [file mmc3.pdf]

Stem Cell Reports
Report
OPEN ACCESS

Salt-inducible kinases transduce mechanical forces into the specification of the pancreatic endocrine lineage

Chenglei Tian,[1,2] Adam Rump,[1,2] Christine Ebeid,[2,3] Anant Mamidi,[2,4] and Henrik Semb[1,2,5,*]
[1]Institute of Translational Stem Cell Research, Helmholtz Diabetes Center, Helmholtz Zentrum Munchen, Munich, Germany
[2]Novo Nordisk Foundation Center for Stem Cell Biology (DanStem), University of Copenhagen, Copenhagen, Denmark
[3]Present address: Novo Nordisk A/S, Copenhagen, Denmark
[4]Present address: FUJIFILM Diosynth Biotechnologies, Copenhagen, Denmark
[5]Lead contact
*Correspondence: henrik.semb@helmholtz-munich.de
https://doi.org/10.1016/j.stemcr.2025.102444

SUMMARY

The extracellular matrix-F-actin-Yes-associated protein 1 (YAP1)-Notch mechanosignaling axis is a gatekeeper in the fate decisions of bi-potent pancreatic progenitors (bi-PPs). However, the link between F-actin dynamics and YAP1 activity remains poorly understood. Here, we identify salt-inducible kinases (SIKs) as mediators of F-actin-triggered changes in YAP1 activity. Interestingly, sodium chloride treatment promotes the differentiation of bi-PPs into NEUROG3$^+$ endocrine progenitors (EPs) through enhanced SIK expression. Consistently, the pan-SIK inhibitor HG-9-09-01 (HG) inhibits latrunculin B (LatB)-induced EP differentiation via nuclear YAP1 accumulation. Unexpectedly, withdrawal of HG after a 12-h treatment increased SIK expression by a negative feedback mechanism, leading to significantly enhanced endocrinogenesis. Therefore, the combined treatment of bi-PPs with LatB and HG for 12 h boosted endocrinogenesis, ultimately leading to an increased number of beta cells. In summary, we identify SIKs as new transducers of mechanotransduction-triggered induction of pancreatic endocrine cell fates.

INTRODUCTION

Type 1 diabetes (T1D) is an autoimmune disease in which beta cells are selectively destroyed, leading to severe insulin deficiency that requires daily insulin injections for survival (Atkinson et al., 2014). Theoretically, human pluripotent stem cells (hPSCs) serve as an unlimited source of functional beta cells, offering a promising advancement in therapy for the growing number of patients with T1D (Schroeder, 2012). The differentiation of beta cells from hPSCs relies on the precise activation and repression of specific developmental pathways by recombinant proteins and chemicals, guiding hPSCs stepwise through stages such as definitive endoderm, primitive gut tube, posterior foregut, bi-potent pancreatic progenitor (bi-PP), endocrine progenitor (EP), and endocrine cell stages (Pagliuca et al., 2014; Rezania et al., 2014; Russ et al., 2015). Among these stages, PDX1$^+$NKX6.1$^+$ bi-PPs have the potential to differentiate into either ductal or endocrine cell lineages. While most established protocols can consistently and efficiently produce bi-PPs *in vitro*, the efficiency of further differentiation from bi-PPs to EPs is imperfect due to lack of full mechanistic comprehension of how this lineage decision is controlled (Ameri et al., 2017; Hogrebe et al., 2020; Mamidi et al., 2018; Pagliuca et al., 2014; Rezania et al., 2014; Russ et al., 2015).

We previously demonstrated that F-actin dynamics, e.g., in response to cell-extracellular matrix (ECM) adhesion, autonomously controls the fate of bi-PPs through Yes-associated protein 1 (YAP1)-Notch mechanosignaling. Specifically, actin depolymerization decreases nuclear YAP1 levels, leading to reduced HES1 expression and increased EP differentiation (Mamidi et al., 2018). Consistent with our findings, Hogrebe et al. reduced F-actin levels in stem cell-derived bi-PPs by latrunculin A treatment, thereby promoting endocrinogenesis and beta cell formation (Hogrebe et al., 2020; Siehler et al., 2020). However, the mechanism by which F-actin regulates nuclear YAP1 activity in bi-PPs remains unknown. Here, we identify salt-inducible kinases (SIKs), members of the AMP-activated protein kinase family, as mediators of the F-actin-triggered effects on YAP1 activity during lineage specification of bi-PPs (Wang et al., 2020). Reduction of F-actin levels by latrunculin B (LatB) increases SIK expression, which diminishes nuclear levels of YAP1 and increases EP differentiation. Consistently, pharmacologically increased SIK expression promotes endocrine cell differentiation and generates more beta cells both *in vitro* (hPSC differentiation) and *ex vivo* (mouse fetal explants). Altogether, these studies reveal the role of SIKs in controlling the fate decision of bi-PPs and provide a valuable new way to increase the conversion of hPSC-derived bi-PPs into endocrine cells.

RESULTS

The pro-endocrinogenic effect of actin depolymerization is mediated by SIKs

To explore the molecular mechanism by which actin depolymerization triggers endocrine cell differentiation,

Published by Elsevier Inc. on behalf of International Society for Stem Cell Research.
This is an open access article under the CC BY license (http://creativecommons.org/licenses/by/4.0/).

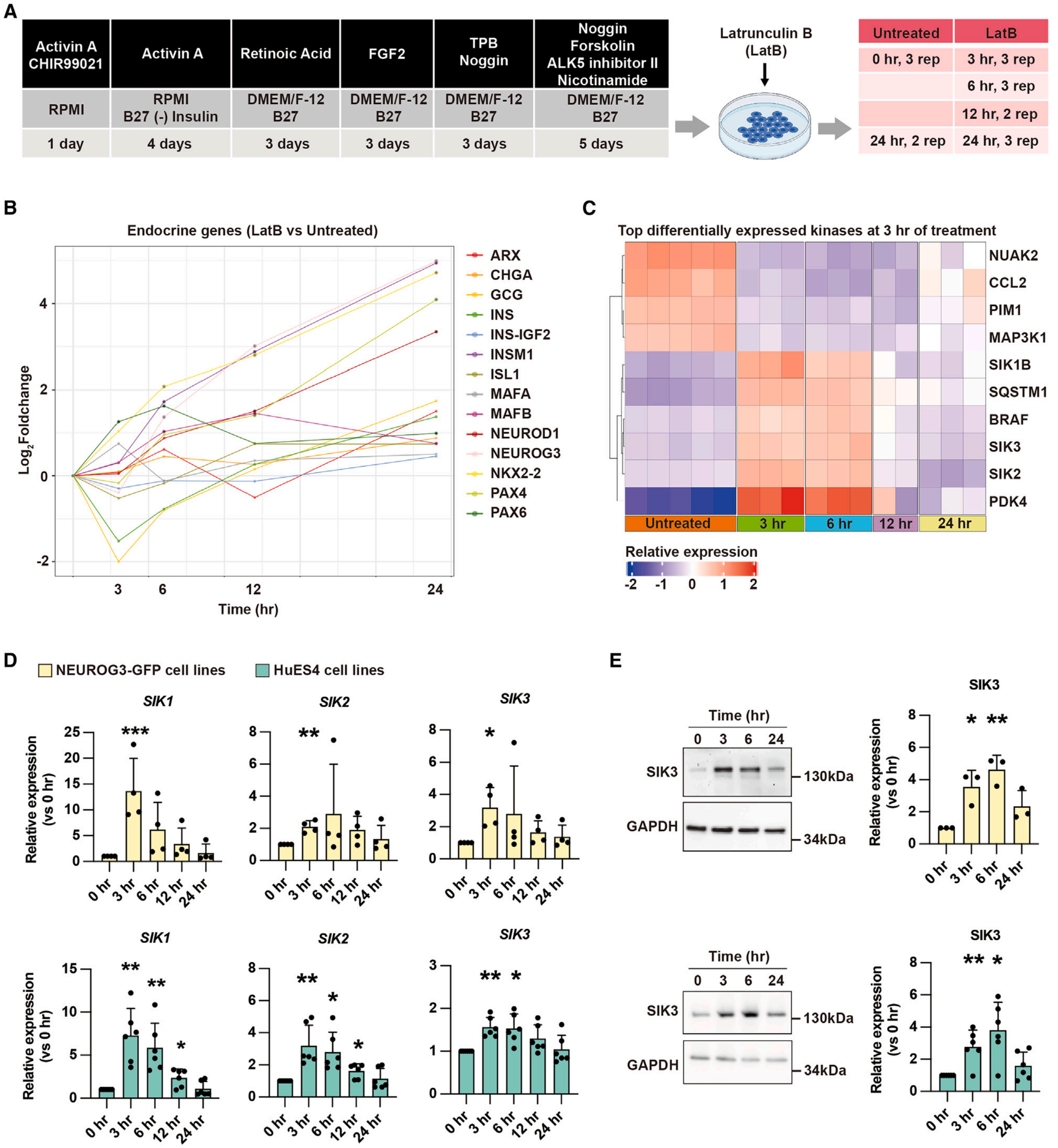

Figure 1. Salt-inducible kinases are induced by latrunculin B in pancreas progenitors

(A) Experimental setup for using bulk RNA-seq to assess the potential downstream targets of F-actin in endocrine cell differentiation. The differentiation protocol generates pancreatic bipotent progenitors (bi-PPs) by day 19. At day 19, latrunculin B (LatB), an actin depolymerization drug, is added to the culture. Samples are then collected at 0, 3, 6, 12, and 24 h post treatment for bulk RNA-seq analysis.

(B) RNA-seq-based time-course analysis of expression of pancreatic endocrine cell markers after LatB treatment. Most of these markers are upregulated at 6 h post LatB treatment. The samples are derived from the NEUROG3-GFP cell lines.

(C) Top differentially expressed kinase-coding genes between the 3 h LatB treatment and untreated samples (relative expression >2, adjusted p value < 0.05). The samples are derived from the NEUROG3-GFP cell lines.

(legend continued on next page)

we treated PDX1$^+$NKX6.1$^+$ bi-PPs with the actin depolymerization drug LatB on day 19, as previously done (Mamidi et al., 2018) (Figures 1A, S1A, and S1B). To precisely identify the F-actin-targeted genes responsible for triggering EP differentiation, we collected samples at multiple time points (3, 6, 12, and 24 h) following LatB treatment for bulk RNA-seq analysis. Untreated samples at 0 and 24 h were used as controls (Figure 1A). Analysis of endocrine cell marker gene expression revealed that early EP marker genes, including *NEUROG3*, *INSM1*, *ISL1*, and *NEUROD1*, increased 6 h post LatB treatment (Figure 1B; Table S1). Therefore, our analysis focused on the 3-h post-LatB treatment time point.

Surprisingly, the most significantly upregulated gene (by adjusted p value) 3 h post LatB treatment was the protein kinase *SIK2*. Further analysis of the top 10 differentially regulated kinases at this time point revealed a significant transient increased expression of *PDK4*, *SQSTM1*, *BRAF*, *SIK1B*, *SIK2*, and *SIK3* (Figure 1C). Notably, none of these kinases have been studied in the context of pancreatic endocrinogenesis. All three SIK family members (*SIK1B*, *SIK2*, and *SIK3*) in the top 10 gene list suggest that SIKs may strongly link to endocrinogenesis. qPCR analysis validated the expression of *SIK1B*, *SIK2*, and *SIK3* (Figure 1D). Western blot analysis corroborated the *SIK3* mRNA data by demonstrating that SIK3 protein levels increased 3–6 h post LatB treatment (Figure 1E). These findings suggest that SIKs act downstream of F-actin dynamics during endocrinogenesis.

Sodium chloride treatment promotes endocrinogenesis through increased SIK expression

SIKs can be exogenously activated by sodium homeostasis (Taub et al., 2010). Treatment with 180 mM sodium chloride (NaCl) for 12 h on day 19 promoted *SIK3* expression, while 200 mM NaCl treatment for the same duration promoted both *SIK1* and *SIK3* expression (Figure 2A). Western blot analysis confirmed that the SIK3 protein levels increased after 12 h with 200 mM NaCl treatment (Figure 2B). Based on these results, a 12-h treatment with 200 mM NaCl at day 19 was chosen to test whether increased SIK expression enhanced EP differentiation. 12-h treatment with 200 mM NaCl does not change the cell viability (Table S2). Consistent with the increased expression of *SIK1* and *SIK3*, *NEUROG3* expression also increased following NaCl treatment (Figure 2C). This observation was corroborated by immunofluorescence and flow cytometry, demonstrating an increased proportion and the number of NEUROG3-GFP$^+$ EPs after NaCl treatment (Figures 2D and 2E; Table S3).

To further verify whether NaCl treatment promotes endocrine cell differentiation, NaCl-treated cells were cultured in a standard medium for an additional 4.5 days (until day 24) (Figure 2F). The NEUROG3-GFP reporter could be used to roughly estimate not only the number of EPs but also the total number of endocrine cells (Beydag-Tasoz et al., 2023; Lof-Ohlin et al., 2017). Flow cytometry revealed that the number of NEUROG3-GFP$^+$ endocrine cells was significantly higher in the NaCl-treated cells compared to the untreated cells on day 24 of differentiation (Figure 2G). Consistently, the expression levels of *INS* and *GCG* were upregulated in the NaCl-treated cells compared to the untreated cells on day 24 (Figure 2H). Furthermore, NaCl treatment resulted in a 1.5-fold increase in beta cell number (Figure 2I). Collectively, these results demonstrate that NaCl treatment enhances pancreatic endocrine lineage specification and suggest that increased SIK expression is the underlying mechanism.

SIKs promote endocrine cell differentiation

In our previous work, we demonstrated that the mechanosensory YAP1 inhibits NEUROG3 expression by binding directly to its promoter (Mamidi et al., 2018). To assess whether SIKs promote endocrine cell differentiation via a reduction in nuclear YAP1, we pharmacologically inhibited SIK activity in LatB-treated cells. To account for potential redundancy among SIK family members, we used the pan-SIK inhibitor HG-9-91-01 (HG), which efficiently blocks the ATP-binding sites of all SIK isoforms (Clark et al., 2012). As expected, *NEUROG3* expression levels were lower in LatB+HG-treated cells compared to the cells treated with LatB alone (Figure 3A). This observation is most likely explained by restored nuclear levels of YAP1 upon HG treatment (Figure 3B). Consistently, the expression of YAP1 target genes *CTGF* and *NUAK2* increased following HG treatment (Figure 3C). These results suggest that the pro-endocrinogenic function of SIKs is mediated by reduced nuclear YAP1 levels. In light of the established link between reduced nuclear YAP1 and increased endocrinogenesis (Mamidi et al., 2018; Rosado-Olivieri et al., 2019), these results suggest that the pro-endocrinogenic function of SIKs involves the suppression of nuclear YAP1.

Interestingly, extended HG treatment has been reported to increase SIK transcription, possibly through a negative feedback mechanism (Wang et al., 2020). We confirmed

(D) qPCR-based time-course analysis of *SIK1*, *SIK2*, and *SIK3* expression levels after LatB treatment. Data represent the mean ± SD. The samples are derived from both NEUROG3-GFP (n = 4 independent repeats) and HuES4 (n = 6 independent repeats) cell lines.
(E) Western blot-based time-course analysis of SIK3 protein levels after LatB treatment. Data represent the mean ± SD. The samples are derived from both NEUROG3-GFP (n = 4 independent repeats) and HuES4 (n = 6 independent repeats) cell lines.
*$p < 0.05$; **$p < 0.01$; ***$p < 0.001$ (two-tailed paired Student's t test). See also Figure S1; Table S1.

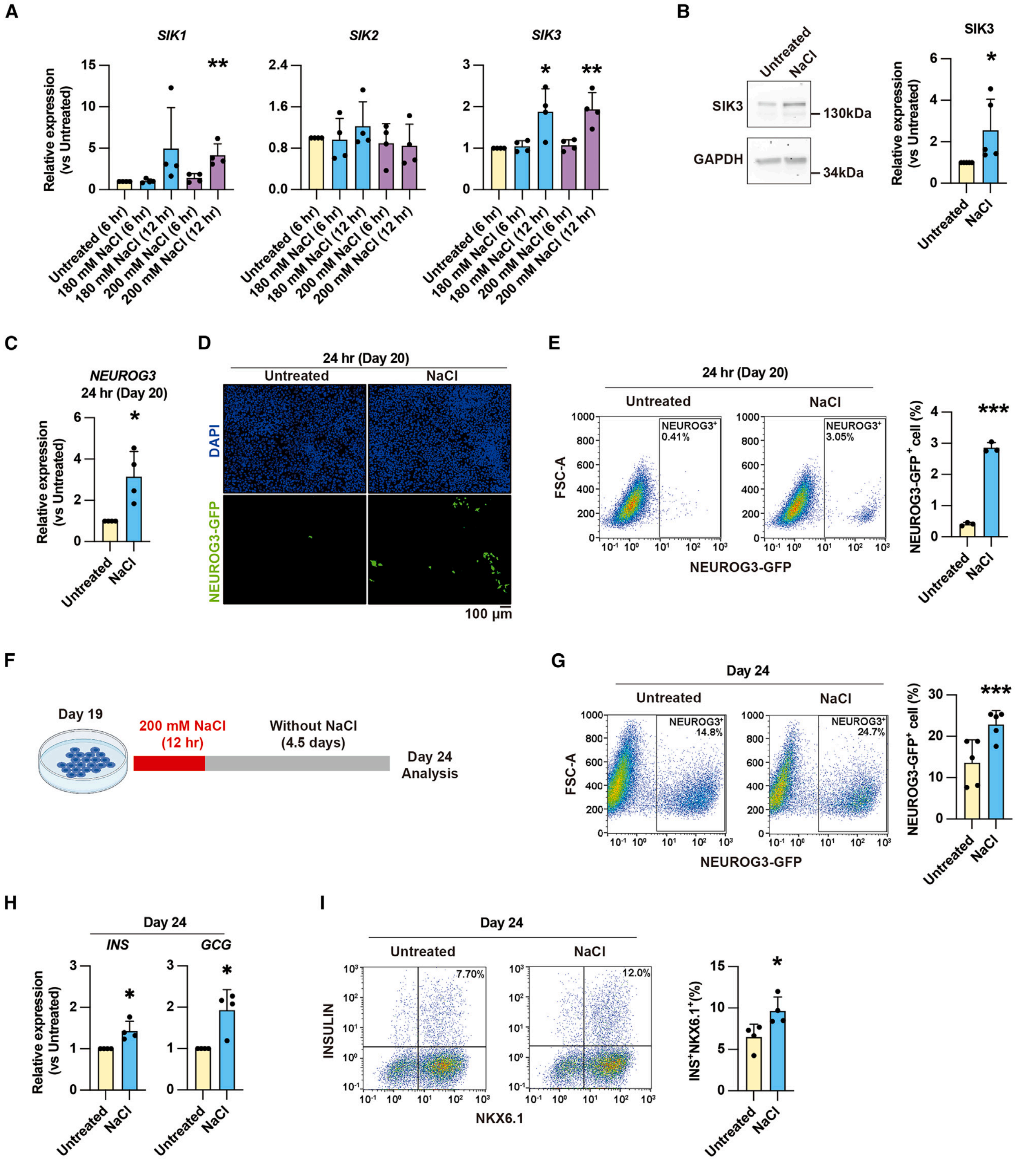

Figure 2. Sodium chloride promotes endocrine cell differentiation by increasing SIK expression

(A) qPCR analysis of *SIK1*, *SIK2*, and *SIK3* expression after 180 and 200 mM sodium chloride (NaCl) treatment for 6 or 12 h. Data represent the mean ± SD (n = 4 independent repeats). The samples are derived from both the HuES4 and NEUROG3-GFP cell lines.

(B) Western blot analysis of SIK3 protein levels after 200 mM NaCl treatment for 12 h. Data represent the mean ± SD (n = 5 independent repeats). The samples are derived from the NEUROG3-GFP cell line.

(legend continued on next page)

this result, as HG treatment alone increased SIK expression (Figure S1C). Consistently, LatB+HG treatment for 12 h significantly increased the mRNA levels of *SIK1* and *SIK3* (Figure 3D). Given that increased SIK expression enhances endocrine cell differentiation, we hypothesized that removing HG after 12 h of treatment would lift the inhibition of SIK activity and enhance endocrine cell differentiation due to increased SIK expression. To test our hypothesis, LatB and HG were removed after 12 h, after which cells were cultured in a standard medium (hereon referred to as 12 h LatB+HG). Western blot analysis showed that the protein levels of SIK3 were significantly higher in LatB+HG-treated cells compared to untreated cells, and these elevated levels persisted for at least 12 h after LatB and HG removal (Figure 3E). As expected, nuclear YAP1 decreased in the 12 h LatB+HG cells compared to the untreated and LatB-treated cells (Figure 3F). Consistently, the expression of EP marker genes *NEUROG3* and *NEUROD1* was significantly higher in the 12 h LatB+HG cells compared to the untreated and LatB-treated cells (Figure 3G). As a result, the number of NEUROG3-GFP$^+$ endocrine cells was significantly higher in the 12 h LatB+HG cells by day 24 of differentiation compared to the untreated and LatB-treated cells (Figure 3H; Tables S2 and S3). Consistent with the effects of 12 h LatB+HG treatment, HG treatment alone for 12 h also increases the expression of EP marker genes *NEUROG3* and *NEUROD1* at day 20, as well as the generation of NEUROG3-GFP$^+$ endocrine cells at day 24 (Figures S1D–S1F; Table S3). Altogether, these data suggest that SIKs are required for endocrine differentiation.

To substantiate our *in vitro* findings *in vivo*, we treated E11.5 pancreatic explants with LatB+HG for 1 day, followed by LatB+HG removal and 2 days of culture (Figure S2A). The LatB+HG treatment significantly increased the expression levels of *Neurog3*, *Ins*, and *Gcg* compared to untreated controls and elevated *Neurog3* and *Gcg* expression levels compared to LatB treatment (Figure S2B). Consistently, whole-mount immunofluorescence analysis demonstrated that LatB+HG treatment significantly increased INS$^+$ beta cell and GCG$^+$ alpha cell generation (Figures S2C and S2D). The reduction in the Ecad area and associated reduced epithelial branching is likely a consequence of increased differentiation of bi-potent progenitors into NEUROG3$^+$ cells following LatB+HG treatment (Figure S2B). This leads to fewer expandable bi-potent progenitors and a reduced number of Ecad$^+$ progenitors. These NEUROG3$^+$ cells subsequently differentiate into endocrine cells, such as GCG$^+$ alpha cells and INS$^+$ beta cells (Figures S2B and S2C). Similar observations have been made in other mouse models where endocrinogenesis was enhanced, such as in *Yap1* or *Hes1* knockout mice (Jensen et al., 2000; Mamidi et al., 2018). In summary, our findings indicate that increased SIK1 and SIK3 expression promotes endocrinogenesis by reduced levels of nuclear YAP1.

Increased SIK expression promotes the generation of functional beta cells

To investigate whether EPs derived from LatB+HG treatment can further differentiate into functional beta cells, we extended the current differentiation protocol until day 35 (Figure 4A). At day 35, LatB+HG-treated cultures contained more beta cells (25.35% ± 3.43%) compared to untreated (10.75% ± 2.48%) and LatB-treated (20.10% ± 4.37%) cultures (Figures 4A and 4B). As expected, the cells expressed characteristic beta cell markers, including C-peptide, NKX6.1, GCG, PDX1, and NEUROD1 (Figure S3). Additionally, due to an increased number of beta cells in the cultures, the combined treatment of LatB and HG shows more insulin content in the cultures than that in untreated and LatB-treated cultures (Figure 4C). Furthermore, LatB+HG treatment resulted in the formation of beta cells that secreted C-peptide in response to high glucose and exendin-4 (Figures 4D and 4E; Table S4). However, the absence of a significant difference in the glucose stimulation index among the untreated, LatB, and LatB+HG groups suggests that LatB and HG treatments do not improve beta cell maturation

(C) qPCR analysis of *NEUROG3* expression levels at 24 h after 200 mM NaCl treatment for 12 h. Data represent the mean ± SD (n = 4 independent repeats). The samples are derived from both the HuES4 and NEUROG3-GFP cell lines.

(D) Imaging shows an increase in NEUROG3-GFP$^+$ endocrine progenitors generated at 24 h after 12 h of 200 mM NaCl treatment compared to untreated cultures. Scale bar, 100 μm.

(E) Flow cytometry-based quantification of NEUROG3-GFP$^+$ cells at 24 h with and without 200 mM NaCl treatment for 12 h. Data represent the mean ± SD (n = 3 independent repeats).

(F) Experimental setup for assessing endocrine cell differentiation after NaCl treatment: day 19 cells are treated with 200 mM NaCl for 12 h, then NaCl is removed, and cultures are maintained for 4.5 days (until day 24) for analysis.

(G) Flow cytometry-based quantification of NEUROG3-GFP$^+$ cells at day 24 with and without 200 mM NaCl treatment for 12 h. Data represent the mean ± SD (n = 5 independent repeats).

(H) qPCR analysis of *INS* and *GCG* expression levels at day 24 with and without 200 mM NaCl treatment for 12 h. Data represent the mean ± SD (n = 4 independent repeats). The samples are derived from the NEUROG3-GFP cell line.

(I) Flow cytometry-based quantification of INS$^+$NKX6.1$^+$ beta cells at day 24 with and without 200 mM NaCl treatment for 12 h. Data represent the mean ± SD (n = 4 independent repeats). The samples are derived from the NEUROG3-GFP cell line.

*p < 0.05; **p < 0.01; ***p < 0.001 (two-tailed paired Student's t test). See also Tables S2 and S3.

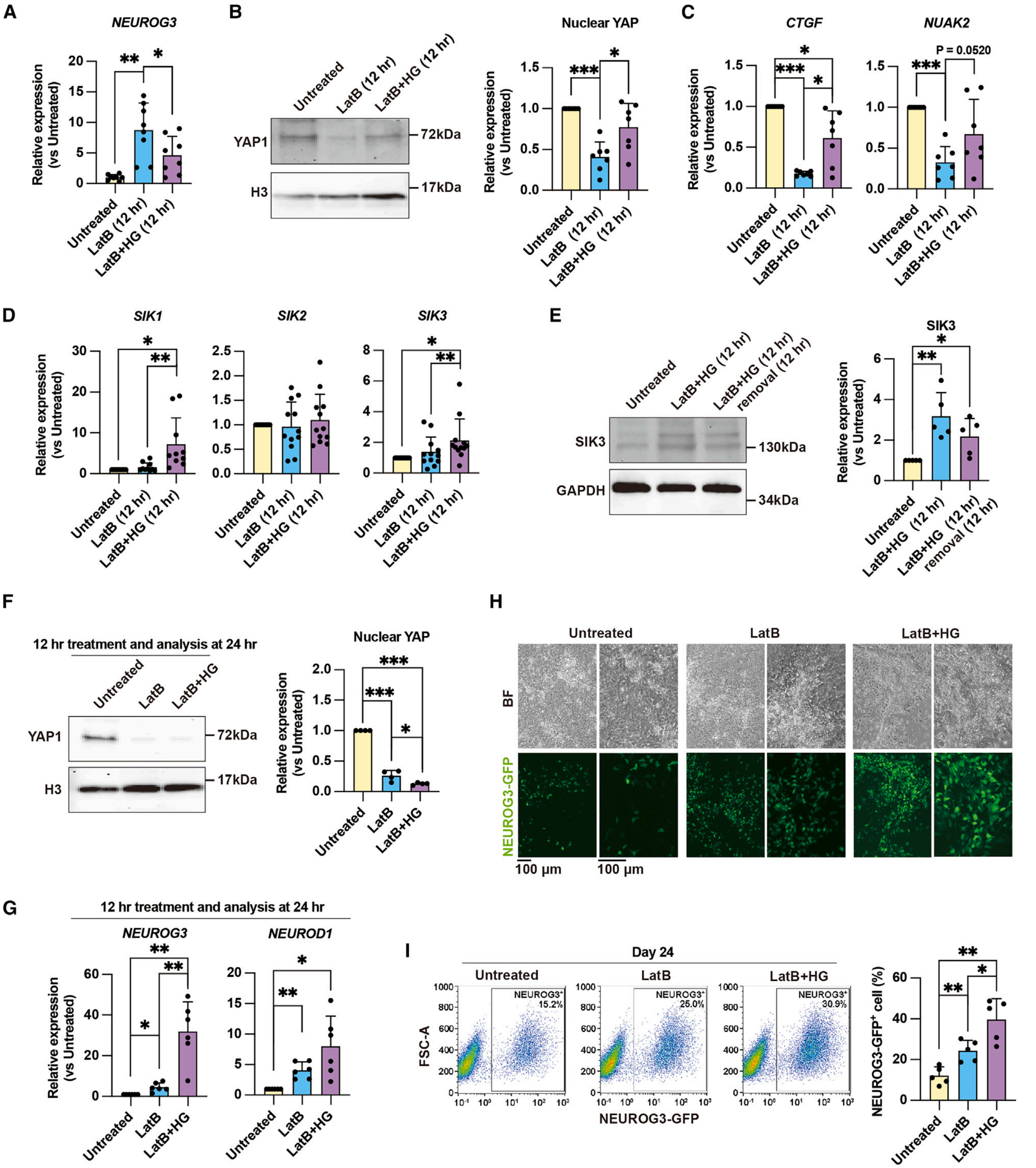

Figure 3. SIKs promote pancreatic endocrine cell differentiation by reducing nuclear YAP

(A) qPCR analysis of *NEUROG3* expression levels at 12 h in untreated, LatB-treated, and LatB+HG-9-91-01 (HG)-treated cells. Data represent the mean ± SD (n = 8 independent repeats). The samples are derived from both the HuES4 and NEUROG3-GFP cell lines.

(B) Western blot analysis of nuclear YAP1 at 12 h in untreated, LatB-treated, and LatB+HG-treated cells. H3 served as a loading control. Data represent the mean ± SD (n = 7 independent repeats). The samples are derived from the NEUROG3-GFP cell line.

(legend continued on next page)

(Table S4). These findings suggest that increased SIK expression following LatB+HG treatment promotes the differentiation of glucose-responsive beta cells.

DISCUSSION

Previous studies demonstrated that changes in ECM interactions control bi-PP fate via the F-actin-YAP1-Notch mechanosignaling axis (Mamidi et al., 2018). However, the exact mechanism by which F-actin dynamics regulate nuclear YAP1 levels remains unclear. Here, we identify SIKs as pivotal downstream mediators of F-actin dynamics in the specification of bi-PPs into EPs. Actin depolymerization induced by LatB increases SIK expression, leading to reduced nuclear YAP1 levels and enhanced NEUROG3 expression (Figure 4F). Notably, pharmacological induction of SIK expression promotes endocrinogenesis and the generation of functional glucose-responsive beta cells.

YAP is a key effector in mechanotransduction (Dupont et al., 2011), governing gene expression changes driven by mechanical signals that shape cell fate decisions during the development of organs, such as the pancreas (Mamidi et al., 2018; Rosado-Olivieri et al., 2019), trophectoderm (Nishioka et al., 2009), kidney (Reginensi et al., 2013), and lung (Mahoney et al., 2014). Furthermore, the link between SIKs and YAP appears to be a widespread phenomenon. For instance, SIK2 and SIK3 regulate the Hippo/YAP pathway in *Drosophila* (Wehr et al., 2013), and SIK1 decreases nuclear YAP levels in mouse and human smooth muscle cells (Pu et al., 2022). These findings suggest that SIKs are conserved regulators of YAP and may play a broader role than previously anticipated in organogenesis and cell fate specification.

SIK expression and activity are influenced by various processes. Here, we demonstrate that mechanical tension, mediated through F-actin dynamics, affects SIK expression. However, the underlying mechanism for this observation remains unclear. One possible explanation is that F-actin may regulate SIK expression by modulating intracellular sodium homeostasis through its effect on ion channels. This hypothesis is supported by evidence that reduction of F-actin levels can increase intracellular Na^+ concentrations via the activation of epithelial Na^+ channels in polarized epithelial cells of the kidney, lung, and colon (Kleyman and Eaton, 2020; Morachevskaya and Sudarikova, 2021). Additionally, SIK activity can be regulated independently of F-actin. For instance, Ca^{2+} modulates intracellular sodium levels through the Na^+/Ca^{2+} exchanger (NCE1) and calcium/calmodulin-dependent protein kinase, increasing or decreasing SIK activity (Sjostrom et al., 2007). Moreover, metabolic stress-induced activation of liver kinase B1 can phosphorylate and activate SIKs (Shackelford and Shaw, 2009). These findings indicate that regulation of metabolic changes or intracellular Na^+ and Ca^{2+} levels in bi-PPs may also enhance endocrinogenesis via increased SIK expression and/or activity.

Bioreactor-based 3D differentiation of hPSCs is currently the most favorable approach for the scalable engineering of pancreatic beta cells. However, directly controlling endocrinogenesis by manipulating the ECM within a 3D system poses challenges. Additionally, the use of actin-depolymerizing drugs, such as LatB, may lead to significant morphological changes or even disaggregation of the cell clusters. Our findings offer alternative strategies for regulating endocrinogenesis that do not rely on ECM components or actin-depolymerizing agents. Here, we show that combined treatment with NaCl and HG boosts endocrinogenesis and beta cell differentiation via enhanced SIK activity, making them promising candidates for similar applications under scalable conditions.

(C) qPCR analysis of *CTGF* and *NUAK2* (YAP target genes) expression levels at 12 h in untreated, LatB-treated, and LatB+HG-treated cells. Data represent the mean ± SD (n = 7 independent repeats). The samples are derived from both the HuES4 and NEUROG3-GFP cell lines.
(D) qPCR analysis of *SIK1/2/3* expression levels at 12 h in untreated, LatB-treated, and LatB+HG-treated cultures. Data represent the mean ± SD (SIK1: n = 10; SIK2 and SIK3: n = 12, independent repeats). The samples are derived from both the HuES4 and NEUROG3-GFP cell lines.
(E) Western blot analysis of SIK3 protein levels at 12 h in untreated and 12 h LatB+HG-treated cultures and 24 h in 12 h LatB+HG + 12 h LatB and HG removal cultures. Data represent the mean ± SD (n = 5 independent repeats). The samples are derived from the NEUROG3-GFP cell line.
(F) Western blot analysis of nuclear YAP at 24 h in untreated, 12 h LatB-treated, and 12 h LatB+HG-treated cultures. H3 served as a loading control. Data represent the mean ± SD (n = 4 independent repeats). The samples are derived from the NEUROG3-GFP cell line.
(G) qPCR analysis of *NEUROG3* and *NEUROD1* expression levels at 24 h in untreated, 12 h LatB-treated, and 12 h LatB+HG-treated cultures. Data represent the mean ± SD (n = 6 independent repeats). The samples are derived from NEUROG3-GFP cell lines.
(H) Imaging shows an increase in NEUROG3-GFP^+ endocrine progenitors generated on day 24 in 12 h LatB+HG-treated cultures compared to untreated or 12 h LatB cultures. Scale bar, 100 μm.
(I) Flow cytometry-based quantification of NEUROG3-GFP^+ cells on day 24 in untreated, 12 h LatB-treated, and 12 h LatB+HG-treated cultures. Data represent the mean ± SD (n = 5 independent repeats).
*$p < 0.05$; **$p < 0.01$; ***$p < 0.001$ (two-tailed paired Student's t test). See also Figures S1 and S2; Tables S2 and S3.

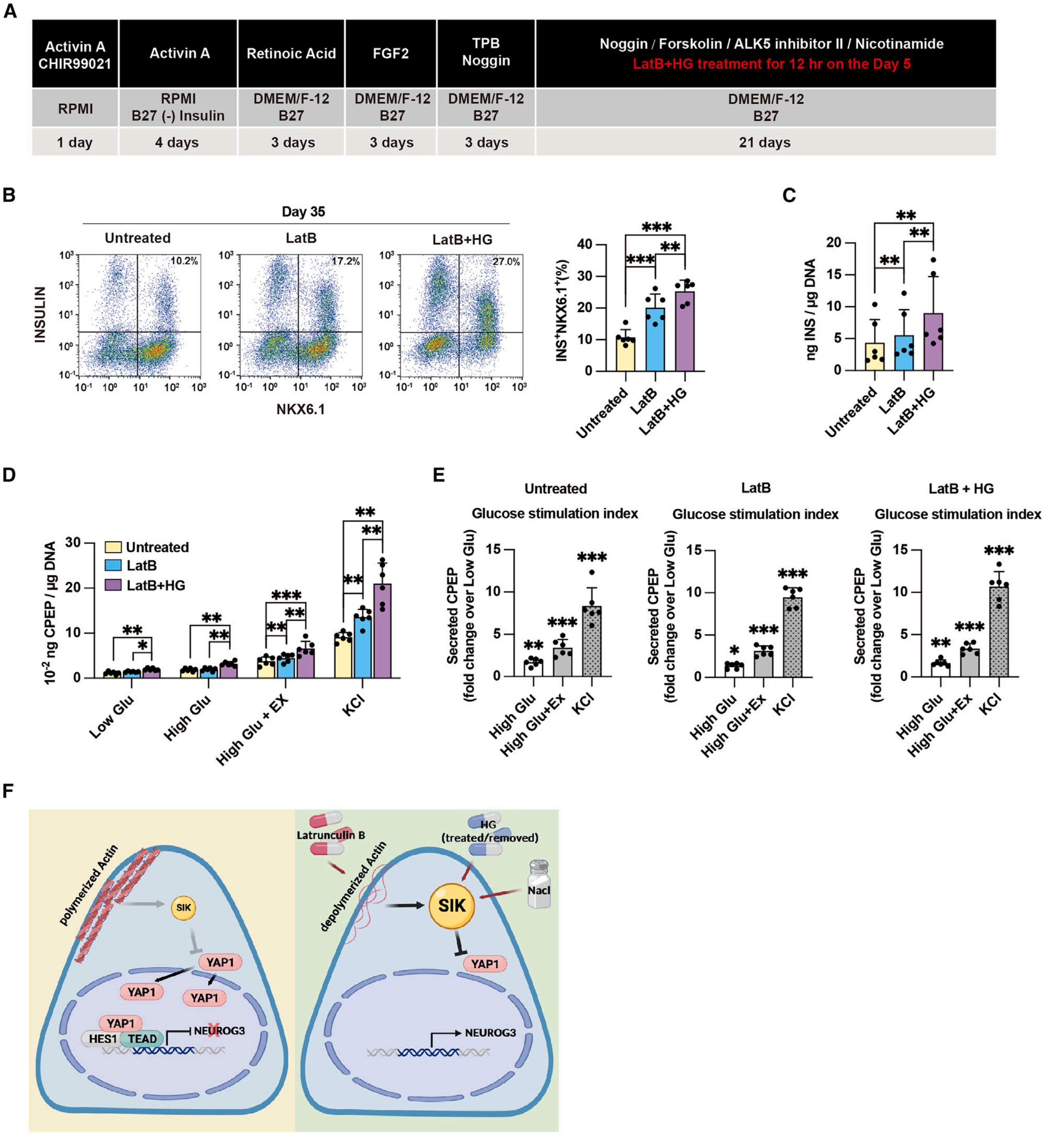

Figure 4. HG promotes functional beta cell generation

(A) Modified protocol for differentiation of hESCs into pancreatic beta cells. At the last stage of differentiation, LatB and HG are added on day 19 of the whole differentiation for 12 h. The cells are collected on day 35 for analysis.

(B) Flow cytometry-based quantification of $INS^{+}NKX6.1^{+}$ beta cells at day 35 in untreated, 12 h LatB-treated, and 12 h LatB+HG-treated cultures. Data represent the mean ± SD (n = 6 independent repeats).

(C) ELISA measurements of total INS content normalized to DNA content in day 35 cells. Data represent the mean ± SD (n = 6 independent repeats).

(legend continued on next page)

In summary, our findings provide valuable new insights into the molecular mechanisms underlying pancreatic endocrine differentiation, emphasizing a crucial role of SIKs as intermediates in the F-actin-YAP1 signaling axis. Additionally, this study introduces SIKs as new targets for boosting beta cell generation from hPSCs.

METHODS

Quantification and statistical analysis

Statistical analyses were performed with GraphPad Prism (version 7.0 or 8.0, GraphPad Software). Unless otherwise noted, a paired nonparametric test (Wilcoxon matched-pairs signed-rank test) was used to assess significance. An unpaired nonparametric test was used for unpaired data (Mann-Whitney test). Asterisks denote p values as follows: *$p < 0.05$; **$p < 0.01$; ***$p < 0.001$. Each N represents a biological replicate (mice fetal pancreas or independent experiments). Data figures illustrate the mean with standard deviation (SD) or with standard error of the mean (SEM) and the values of individual biological replicates.

RESOURCE AVAILABILITY

Lead contact

Further information and requests for resources and reagents should be directed to and will be fulfilled by the lead contact, Henrik Semb (henrik.semb@helmholtz-munich.de).

Materials availability

All unique/stable reagents in this study are available from the lead contact with a completed Materials Transfer Agreement.

Data and code availability

- The accession number for the RNA-seq datasets reported in this paper is GSE275775. They are publicly available as of the date of publication.
- This paper does not report original code.
- Any additional information required to reanalyze the data reported in this paper is available from the lead contact upon request.

ACKNOWLEDGMENTS

We thank Anna Øster for her excellent technical support, Anna Månsson for helping with animal experiments, and Ivan Kulik for assistance with the immunofluorescence. All the work is supported by the European Union's Horizon 2020 research and innovation program (ISLET, no. 874839), the Novo Nordisk Foundation Center for Stem Cell Biology (DanStem) at the University of Copenhagen (NNF grant, NNF17CC0027852), and the Helmholtz Zentrum München.

AUTHOR CONTRIBUTIONS

C.T., A.R., C.E., A.M., and H.S. designed and interpreted the experiments. C.T. performed most of the experiments and analyses. A.R. performed and analyzed RNA-seq data. C.E. performed and analyzed fetal mouse pancreas experiments. A.M. performed some of the *in vitro* differentiation and data analysis. C.T. and H.S. wrote the manuscript.

DECLARATION OF INTERESTS

H.S. is a member of the Stem Cell Reports editorial board.

SUPPLEMENTAL INFORMATION

Supplemental information can be found online at https://doi.org/10.1016/j.stemcr.2025.102444.

Received: October 11, 2024
Revised: February 6, 2025
Accepted: February 6, 2025
Published: March 6, 2025

REFERENCES

Ameri, J., Borup, R., Prawiro, C., Ramond, C., Schachter, K.A., Scharfmann, R., and Semb, H. (2017). Efficient Generation of Glucose-Responsive Beta Cells from Isolated GP2(+) Human Pancreatic Progenitors. Cell Rep. *19*, 36–49. https://doi.org/10.1016/j.celrep.2017.03.032.

Atkinson, M.A., Eisenbarth, G.S., and Michels, A.W. (2014). Type 1 diabetes. Lancet *383*, 69–82. https://doi.org/10.1016/S0140-6736(13)60591-7.

Beydag-Tasoz, B.S., D'Costa, J.V., Hersemann, L., Lee, B.H., Luppino, F., Kim, Y.H., Zechner, C., and Grapin-Botton, A. (2023). Integrating single-cell imaging and RNA sequencing datasets links differentiation and morphogenetic dynamics of human pancreatic

(D) ELISA measurements of secreted C-peptide after 30 min in 1.67 mM glucose (low Glu), 16.67 mM glucose (high Glu), 16.67 mM glucose plus 10 nM exendin-4 (high Glu+Ex), and 1.67 mM glucose with 30 mM KCl (KCl) condition. The C-peptide secretion normalized to DNA content in day 35 cells. Data represent the mean ± SD (n = 6 independent repeats).
(E) The fold change of C-peptide secretion in 30 min high Glu, high Glu+Ex, and KCl relative to low Glu. Asterisks indicate the significance between each condition and low Glu. Data represent the mean ± SD (n = 6 independent repeats).
(F) Model illustrating the proposed mechanism for SIKs transducing F-actin-triggered bi-PP fate decisions. Actin depolymerization increases SIK expression, which reduces nuclear YAP1 levels and increases NEUROG3 expression. Pharmacological induction of SIK expression, through treatments with LatB, NaCl, or HG-9-91-01 (HG), promotes NEUROG3 expression and endocrinogenesis.
*$p < 0.05$; **$p < 0.01$; ***$p < 0.001$ (two-tailed paired Student's t test). The samples are derived from both SA121 (n = 4 independent repeats) and HuES4 (n = 2 independent repeats) cell lines. See also Figure S3; Table S4.

endocrine progenitors. Dev. Cell *58*, 2292–2308.e2296. https://doi.org/10.1016/j.devcel.2023.07.019.

Clark, K., MacKenzie, K.F., Petkevicius, K., Kristariyanto, Y., Zhang, J., Choi, H.G., Peggie, M., Plater, L., Pedrioli, P.G.A., McIver, E., et al. (2012). Phosphorylation of CRTC3 by the salt-inducible kinases controls the interconversion of classically activated and regulatory macrophages. Proc. Natl. Acad. Sci. USA *109*, 16986–16991. https://doi.org/10.1073/pnas.1215450109.

Dupont, S., Morsut, L., Aragona, M., Enzo, E., Giulitti, S., Cordenonsi, M., Zanconato, F., Le Digabel, J., Forcato, M., Bicciato, S., et al. (2011). Role of YAP/TAZ in mechanotransduction. Nature *474*, 179–183. https://doi.org/10.1038/nature10137.

Hogrebe, N.J., Augsornworawat, P., Maxwell, K.G., Velazco-Cruz, L., and Millman, J.R. (2020). Targeting the cytoskeleton to direct pancreatic differentiation of human pluripotent stem cells. Nat. Biotechnol. *38*, 460–470. https://doi.org/10.1038/s41587-020-0430-6.

Jensen, J., Pedersen, E.E., Galante, P., Hald, J., Heller, R.S., Ishibashi, M., Kageyama, R., Guillemot, F., Serup, P., and Madsen, O.D. (2000). Control of endodermal endocrine development by Hes-1. Nat. Genet. *24*, 36–44. https://doi.org/10.1038/71657.

Kleyman, T.R., and Eaton, D.C. (2020). Regulating ENaC's gate. Am. J. Physiol. Cell Physiol. *318*, C150–C162. https://doi.org/10.1152/ajpcell.00418.2019.

Lof-Ohlin, Z.M., Nyeng, P., Bechard, M.E., Hess, K., Bankaitis, E., Greiner, T.U., Ameri, J., Wright, C.V., and Semb, H. (2017). EGFR signalling controls cellular fate and pancreatic organogenesis by regulating apicobasal polarity. Nat. Cell Biol. *19*, 1313–1325. https://doi.org/10.1038/ncb3628.

Mahoney, J.E., Mori, M., Szymaniak, A.D., Varelas, X., and Cardoso, W.V. (2014). The hippo pathway effector Yap controls patterning and differentiation of airway epithelial progenitors. Dev. Cell *30*, 137–150. https://doi.org/10.1016/j.devcel.2014.06.003.

Mamidi, A., Prawiro, C., Seymour, P.A., de Lichtenberg, K.H., Jackson, A., Serup, P., and Semb, H. (2018). Mechanosignalling via integrins directs fate decisions of pancreatic progenitors. Nature *564*, 114–118. https://doi.org/10.1038/s41586-018-0762-2.

Morachevskaya, E.A., and Sudarikova, A.V. (2021). Actin dynamics as critical ion channel regulator: ENaC and Piezo in focus. Am. J. Physiol. Cell Physiol. *320*, C696–C702. https://doi.org/10.1152/ajpcell.00368.2020.

Nishioka, N., Inoue, K.i., Adachi, K., Kiyonari, H., Ota, M., Ralston, A., Yabuta, N., Hirahara, S., Stephenson, R.O., Ogonuki, N., et al. (2009). The Hippo signaling pathway components Lats and Yap pattern Tead4 activity to distinguish mouse trophectoderm from inner cell mass. Dev. Cell *16*, 398–410. https://doi.org/10.1016/j.devcel.2009.02.003.

Pagliuca, F.W., Millman, J.R., Gürtler, M., Segel, M., Van Dervort, A., Ryu, J.H., Peterson, Q.P., Greiner, D., and Melton, D.A. (2014). Generation of functional human pancreatic beta cells in vitro. Cell *159*, 428–439. https://doi.org/10.1016/j.cell.2014.09.040.

Pu, J., Wang, F., Ye, P., Jiang, X., Zhou, W., Gu, Y., and Chen, S. (2022). Salt-inducible kinase 1 deficiency promotes vascular remodeling in pulmonary arterial hypertension via enhancement of yes-associated protein-mediated proliferation. Heliyon *8*, e11016. https://doi.org/10.1016/j.heliyon.2022.e11016.

Reginensi, A., Scott, R.P., Gregorieff, A., Bagherie-Lachidan, M., Chung, C., Lim, D.S., Pawson, T., Wrana, J., and McNeill, H. (2013). Yap- and Cdc42-dependent nephrogenesis and morphogenesis during mouse kidney development. PLoS Genet. *9*, e1003380. https://doi.org/10.1371/journal.pgen.1003380.

Rezania, A., Bruin, J.E., Arora, P., Rubin, A., Batushansky, I., Asadi, A., O'Dwyer, S., Quiskamp, N., Mojibian, M., Albrecht, T., et al. (2014). Reversal of diabetes with insulin-producing cells derived in vitro from human pluripotent stem cells. Nat. Biotechnol. *32*, 1121–1133. https://doi.org/10.1038/nbt.3033.

Rosado-Olivieri, E.A., Anderson, K., Kenty, J.H., and Melton, D.A. (2019). YAP inhibition enhances the differentiation of functional stem cell-derived insulin-producing beta cells. Nat. Commun. *10*, 1464. https://doi.org/10.1038/s41467-019-09404-6.

Russ, H.A., Parent, A.V., Ringler, J.J., Hennings, T.G., Nair, G.G., Shveygert, M., Guo, T., Puri, S., Haataja, L., Cirulli, V., et al. (2015). Controlled induction of human pancreatic progenitors produces functional beta-like cells in vitro. EMBO J. *34*, 1759–1772. https://doi.org/10.15252/embj.201591058.

Schroeder, I.S. (2012). Potential of pluripotent stem cells for diabetes therapy. Curr. Diabetes Rep. *12*, 490–498. https://doi.org/10.1007/s11892-012-0292-5.

Shackelford, D.B., and Shaw, R.J. (2009). The LKB1-AMPK pathway: metabolism and growth control in tumour suppression. Nat. Rev. Cancer *9*, 563–575. https://doi.org/10.1038/nrc2676.

Siehler, J., Blöchinger, A.K., and Lickert, H. (2020). Pharmacological Targeting of the Actin Cytoskeleton to Drive Endocrinogenesis. Trends Pharmacol. Sci. *41*, 384–386. https://doi.org/10.1016/j.tips.2020.04.002.

Sjostrom, M., Stenstrom, K., Eneling, K., Zwiller, J., Katz, A.I., Takemori, H., and Bertorello, A.M. (2007). SIK1 is part of a cell sodium-sensing network that regulates active sodium transport through a calcium-dependent process. Proc. Natl. Acad. Sci. USA *104*, 16922–16927. https://doi.org/10.1073/pnas.0706838104.

Taub, M., Springate, J.E., and Cutuli, F. (2010). Targeting of renal proximal tubule Na,K-ATPase by salt-inducible kinase. Biochem. Biophys. Res. Commun. *393*, 339–344. https://doi.org/10.1016/j.bbrc.2010.02.037.

Wang, C., Song, D., Fu, J., and Wen, X. (2020). SIK1 Regulates CRTC2-Mediated Gluconeogenesis Signaling Pathway in Human and Mouse Liver Cells. Front. Endocrinol. *11*, 580. https://doi.org/10.3389/fendo.2020.00580.

Wehr, M.C., Holder, M.V., Gailite, I., Saunders, R.E., Maile, T.M., Ciirdaeva, E., Instrell, R., Jiang, M., Howell, M., Rossner, M.J., and Tapon, N. (2013). Salt-inducible kinases regulate growth through the Hippo signalling pathway in Drosophila. Nat. Cell Biol. *15*, 61–71. https://doi.org/10.1038/ncb2658.

Stem Cell Reports, Volume *20*

Supplemental Information

Salt-inducible kinases transduce mechanical forces into the specification of the pancreatic endocrine lineage

Chenglei Tian, Adam Rump, Christine Ebeid, Anant Mamidi, and Henrik Semb

Supplemental Information

Salt-inducible kinases transduce mechanical forces into the specification of the pancreatic endocrine lineage

Chenglei Tian, Adam Rump, Christine Ebeid, Anant Mamidi, Henrik Semb

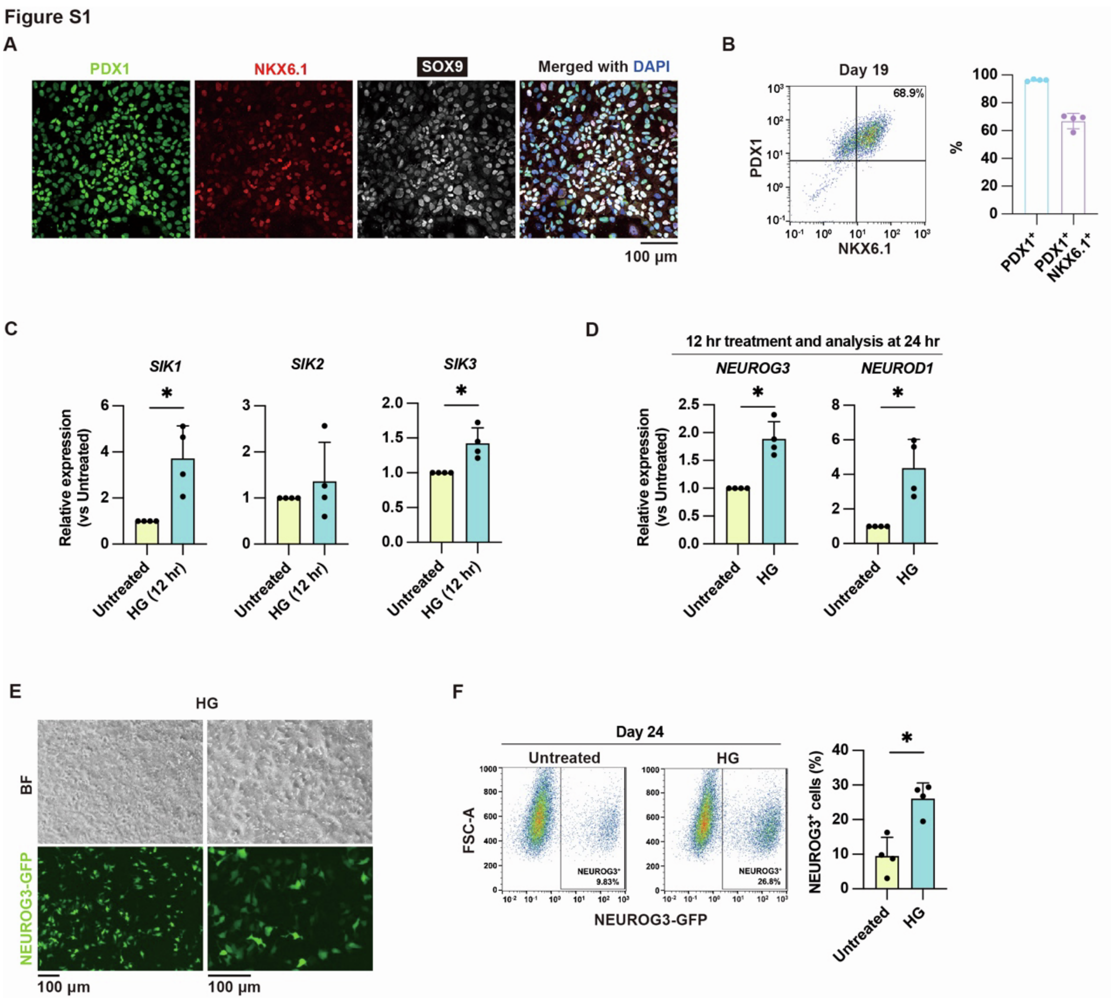

Supplementary Figure 1. Identification of bi-potent pancreatic progenitors (bi-PPs) at Day 19 and HG increases *SIK1* and *SIK3* expression, related to Figures 1 and 3.

(A) Typical confocal images of PDX1, NKX6.1, and SOX9 expression in D19 hESC-derived bi-PPs. Scale bar, 100 μm. The samples are derived from the HuES4 cell line.

(B) Flow cytometry-based quantification of PDX^{+}NKX6.1^{+} in D19 hESC-derived bi-PPs. Data represent the mean ± SD (n = 4 independent repeats). The samples are derived from the HuES4 cell line.

(C) qPCR analysis of *SIK1*, *SIK2*, and *SIK3* expression levels at 12 hr in untreated and HG-treated cultures. Data represent the mean ± SD (n = 4 independent repeats). The samples are derived from the NEUROG3-GFP cell line.

(D) qPCR analysis of *NEUROG3* and *NEUROD1* expression levels at 24 hr in untreated and 12 hr HG-treated cultures. Data represent the mean ± SD (n = 4 independent repeats). The samples are derived from NEUROG3-GFP cell lines.

(E) Imaging shows NEUROG3-GFP^{+} endocrine progenitors generated on Day 24 in 12 hr HG-treated cultures. Scale bar, 100 μm.

(F) Flow cytometry-based quantification of NEUROG3-GFP^{+} cells on Day 24 in untreated and 12 hr HG-treated cultures. Data represent the mean ± SD (n = 4 independent repeats).

*, $p<0.05$ (two-tailed paired Student’s t-test).

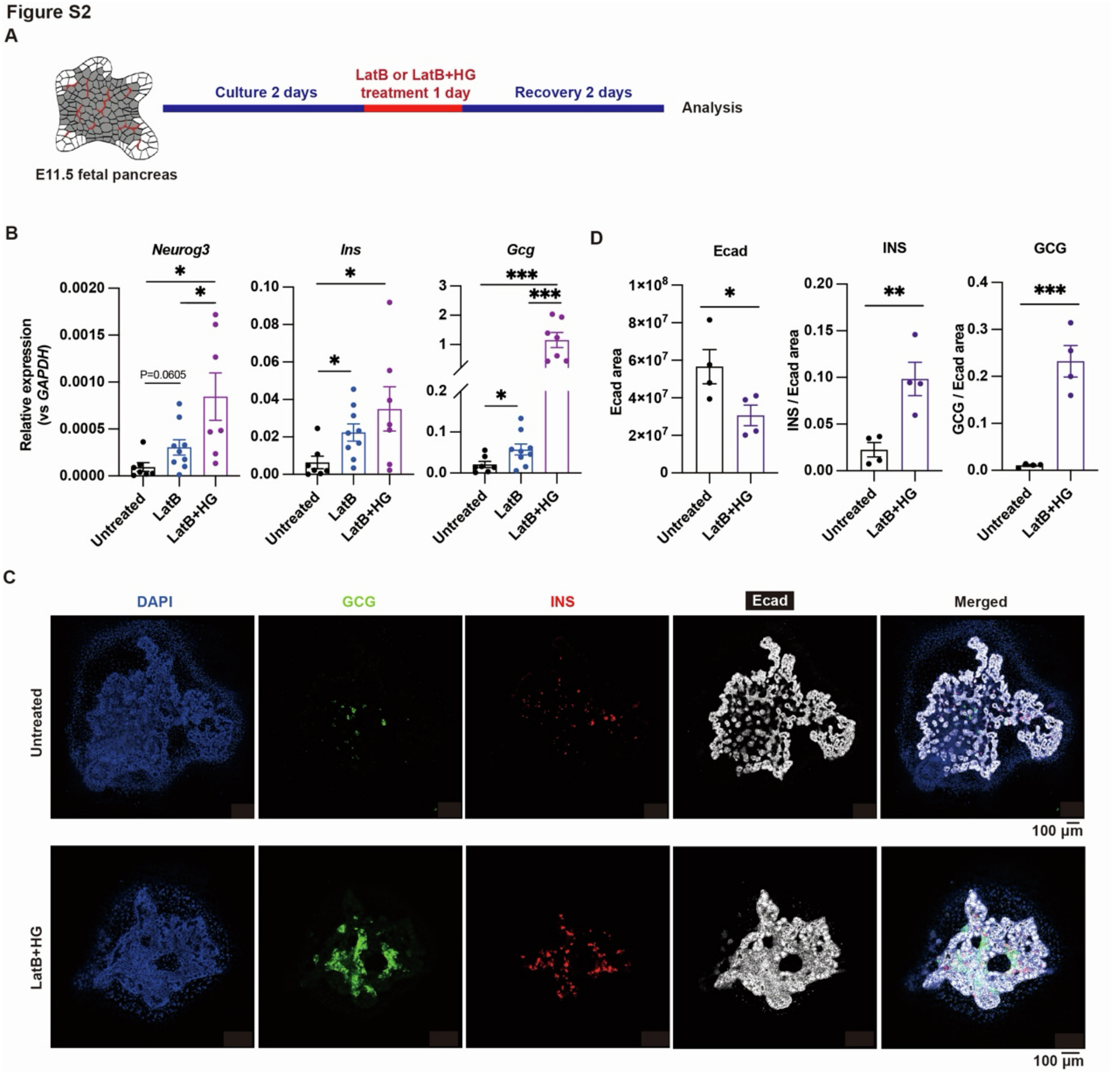

Supplementary Figure 2. LatB+HG promotes endocrine cell differentiation in e*x vivo* mouse embryonic pancreas, related to Figure 3.

(A) Experimental setup for assessing endocrine cell differentiation after LatB+HG treatment in mouse embryonic pancreas: E11.5 pancreata are cultured for 2 days, treated with LatB or LatB+HG for 1 day, and then cultured for an additional 2 days without treatment before analysis.

(B) qPCR analysis of *Neurog3*, *Ins*, and *Gcg* expression in untreated, LatB treated, and LatB+HG-treated fetal pancreas. Data represent the mean ± SEM (Untreated and LatB+HG: n = 7 fetal pancreas; LatB: n = 9 fetal pancreas).

(C) Typical whole-mount immunostaining of GCG, INS, and Ecad expression in *ex vivo* cultured embryonic pancreas. Scale bar, 100 μm.

(D) Image-based quantification of E-cadherin (Ecad)$^{+}$, INS^{+} or GCG^{+} area in untreated and LatB+HG-treated *ex vivo* embryonic pancreas. Ecad, which marks all epithelial areas, is used for normalization. Data represent the mean ± SD (n = 4 fetal pancreas).

*, p<0.05; **, p<0.01; ***, p<0.001 (the unpaired two-samples Wilcoxon test).

Figure S3

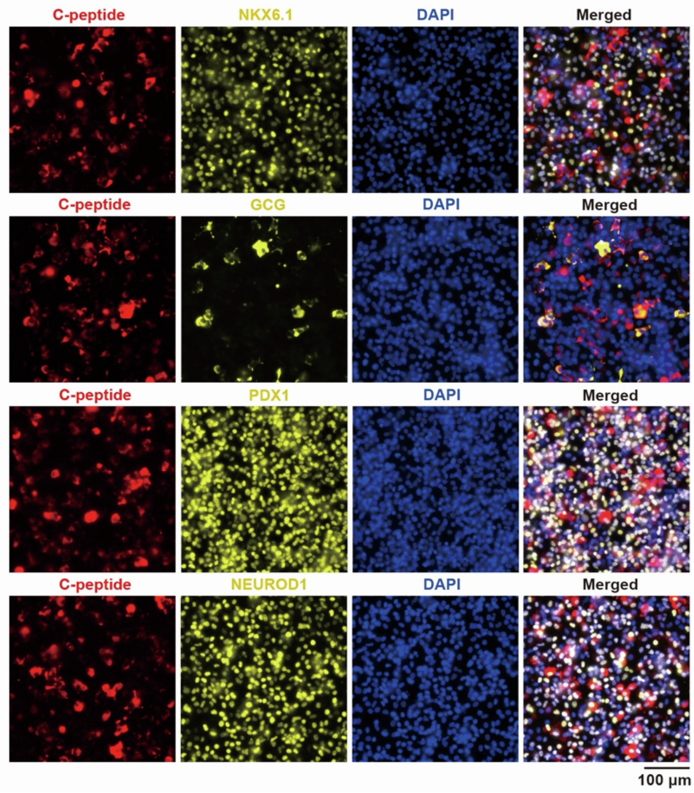

Supplementary Figure 3. Immunofluorescence analysis of C-peptide, NKX6.1, GCG, PDX1, and NEUROD1 expression in LatB+HG-treated D35 cultures, related to Figure 4. Scale bar, 100 μm. The samples are derived from the SA121 cell line.

Figure S4

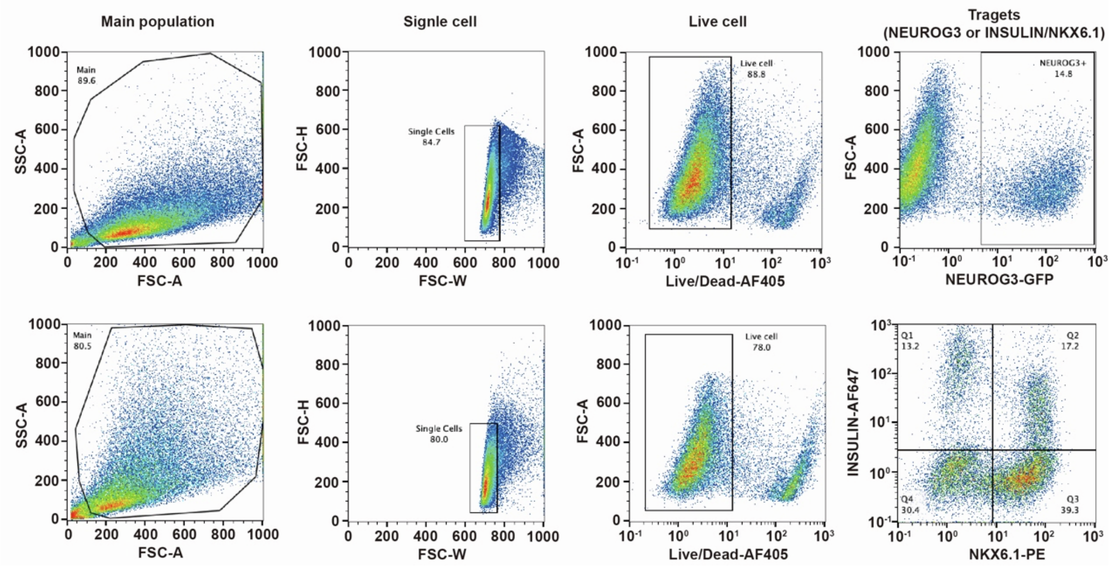

Supplementary Figure 4. Examples of FACS gating strategy, related to Figures 2, 3, and 4.

Supplementary Table 1. Summary of differentially expressed genes, KEGG, and the Gene Ontology analysis at each time point, related to Figure 1.

Supplementary Table 2. Summary of the cell viability by Live/Dead staining after 12 hr treatment, related to Figures 2 and 3.

	Untreated	200 mM NaCl	LatB	HG	LatB + HG
Viability (%)	88.58±7.97	86.85±8.85	76.60±13.01	83.45±9.63	72.83±11.27*

Asterisks indicate the significance between each condition and Untreated. Data represent the mean ± SD (n = 4 independent repeats).

*, p<0.05 (two-tailed paired Student's t-test).

Supplementary Table 3. Summary of total cell and NEUROG3-GFP$^+$ cell numbers on Day 24, related to Figures 2, 3 and 4.

	Untreated	200 mM NaCl	LatB	HG	LatB + HG
Total cell number (10^6/12-well)	1.40±0.12	1.09±0.20	1.06±0.27*	0.96±0.24	0.67±0.10**
NEUROG3-GFP$^+$ cell number (10^6/12-well)	0.13±0.07	0.18±0.06	0.25±0.05	0.24±0.05*	0.27±0.05*

Asterisks indicate the significance between each condition and Untreated. Data represent the mean ± SD (n = 4 independent repeats).

*, p<0.05; **, p<0.01 (two-tailed paired Student's t-test).

Supplementary Table 4. Summary of the fold change of C-peptide secretion in 30 minutes High Glu, High Glu+Ex, and KCl relative to Low Glu in all the groups, related to Figure 4.

	Untreated	LatB	LatB + HG
High Glu	1.71±0.43**	1.37±0.32*	1.65±0.36**
High Glu + Ex	3.41±0.98***	3.13±0.58***	3.37±0.61***
KCl	8.35±2.14***	9.49±1.10***	10.69±1.76***

Asterisks indicate the significance between each condition and Low Glu. Data represent the mean ± SD (SA121 cell line: n = 4 and HuES4 cell line: n = 2, independent repeats).

*, p<0.05; **, p<0.01; ***, p<0.001 (two-tailed paired Student's t-test).

Supplemental Experimental Procedures

KEY RESOURCES TABLE

REAGENT or RESOURCE	SOURCE	IDENTIFIER
**Antibodies**		
Alexa fluor 647 anti-C-peptide	BD Pharmingen	# 565831<br>RRID: AB_2739371
Alexa fluor 647 anti-INSULIN	BD Pharmingen	#565689<br>RRID: AB_2739331
PE anti-NKX6.1	BD Pharmingen	#563023<br>RRID: AB_2716792
GLUCAGON	Cell Signaling	#8233<br>RRID: AB_10859908
PDX1	R&D Systems	#AF2419<br>RRID: AB_355257
NKX6.1	DSHB	#F55A12<br>RRID: AB_532379
SOX9	Sigma Aldrich	#HPA001758<br>RRID: AB_1080067
NEUROD1	Proteintech	#12081-1-AP<br>RRID: AB_2877823
GAPDH	R&D Systems	#AF5718<br>RRID: AB_2278695
H3	Abcam	#ab1791<br>RRID: AB_302613
YAP1	Cell Signaling	#14074<br>RRID: AB_2650491
SIK3	Abcam	#ab88495<br>RRID: AB_2042747
SIK3	Cell Signaling	#39477<br>RRID: AB_3251492
**Chemicals, peptides, and recombinant proteins**		
CHIR99021	Axon Medchem	#1386
Activin A	PeproTech	#120-14
FGF2	PeproTech	#100-18B
Retinoic acid	Sigma Aldrich	#R2625
TBP	Calbiochem	#565740
ALK5iII	Santa Cruz	#sc-221234A
Noggin	PeproTech	#120-10C
Nicotinamide	Sigma Aldrich	#481907
Forskolin	Sigma Aldrich	#F6886
HG-9-91-01	Selleckchem	#S8393
Latrunculin B	Sigma Aldrich	#L5288
Bovine serum albumin (BSA)	Sigma Aldrich	#B4287
Y-27632	Merck Millipore	#688000
Biolaminin 521 LN (LN521)	Bio Lamina	#LN521-05
B27 supplement minus Insulin	Thermo Fisher	#A1895601
B27 supplement	Thermo Fisher	#17504001
Sodium Chloride (NaCl)	Sigma Aldrich	#S3014
Exendin-4	Sigma Aldrich	#E7144
**Critical commercial assays**		

RNeasy Mini Kit	Qiagen	#74106
iScript cDNA Synthesis Kit	BIO-RAD	#1708891
Subcellular Protein Fractionation Kits	Thermo Fisher	#78840
LIVE/DEAD^{TM} Fixable Violet Dead Cell Stain Kit	Thermo Fisher	#L34964
Ultrasensitive C-peptide ELISA	Mercodia	#10-1141-01
Insulin ELISA	Mercodia	#10-1113-10
**Deposited data**		
RNA-seq datasets	This paper	GSE275775
**Experimental models: Cell lines**		
SA121	Takara bio	BioSamples: SAMEA6407434
NEUROG3-GFP (SA121)	Zarah M. Löf-Öhlin, et al (Lof-Ohlin et al., 2017)	NA
HuES4	Harvard University	BioSamples: SAMEA114214019
**TaqMan^{TM} probes**		
GAPDH	Thermo Fisher	Hs02758991
HPRT1	Thermo Fisher	Hs01102345
RPL37A	Thermo Fisher	Hs99999909
SIK1	Thermo Fisher	Hs02573456
SIK2	Thermo Fisher	Hs01568566
SIK3	Thermo Fisher	Hs00228549
NEUROG3	Thermo Fisher	Hs01875204
NEUROD1	Thermo Fisher	Hs00159598
INS	Thermo Fisher	Hs02741908
GCG	Thermo Fisher	Hs01031536
Gapdh	Thermo Fisher	Mm99999915
Neurog3	Thermo Fisher	Mm00437606
Ins	Thermo Fisher	Mm01950294
Gcg	Thermo Fisher	Mm00801714
**Experimental models: Organisms/strains**		
C57BL/6 wildtype mice	In house	N/A
**Software and algorithms**		
Adobe Photoshop 2022	Adobe	https://www.adobe.com
Adobe Illustrator 2023	Adobe	https://www.adobe.com
Fiji 2.0/ImageJ	NIH Image	http://imagej.nih.gov/ij
GraphPad Prism 10	GraphPad	https://www.graphpad.com
FlowJo 10	BD	https://www.flowjo.com
RStudio	RStudio	https://posit.co/downloads/

The use of human embryonic stem cell (hESC) lines

SA121 (XY, RRID: CVCL_B296), NEUROG3-GFP (generated based on SA121 hESC line), and HuES4 (XY, RRID: CVCL_B172) hESC lines were used in this paper. SA121 cell line is from Takara Bio (Y00020), and the HuES4 line is from Harvard University. The karyotype of

hESCs was normal. All hESC lines were cultured on Biolaminin 521 LN (LN521) according to the manufacturer's protocol with daily changes of NutriStem® hPSC XF Medium (Sartorius, # 05-100-1A) at 37 °C and 5% CO_2. Cells were passaged twice to three times (seeding as 40,000 -60,000 cells/cm^2) weekly at subconfluency using StemPro™ Accutase™ (Thermo Fisher, #A1110501). The medium was supplemented with 10 µM ROCK inhibitor (Y-27632) on the first day post-passaging. hESCs were frozen by CryoStor cryopreservation media (Sigma, #C2874). hESCs were sterile and tested negative for mycoplasma in regular tests. All experiments were conducted using cells within 2 to 10 passages after thawing.

The use of animals

Mice were housed at the University of Copenhagen, and all experiments were performed according to guidelines and ethics approved by the Danish Animal Experiments Inspectorate (Dyreforsøgstilsynet). Data were collected from both male and female embryos.

hESC differentiation

hESCs were re-seeded on LN521 for pancreatic differentiation at 80,000 cells/cm^2. At 100% confluency, cells were induced to be further differentiated into pancreatic lineages following the Ameri et al. protocol (Ameri et al., 2017). For Latrunculin B (LatB) treatment, 0.5 µM LatB was added to the protocol for up to 24 hours on Day 19 to enhance endocrine differentiation (Mamidi et al., 2018). For NaCl treatment, since DMEM/F12 medium (Thermo Fisher, #11320033) contains ~120 mM NaCl, 60 mM (180 mM NaCl totally in the medium) or 80 mM (200 mM NaCl totally in the medium) NaCl was added to the protocol for 6 or 12 hours on Day 19. For LatB+HG-9-91-01 (HG) treatment, 4 µM HG was added alone or plus 0.5 µM LatB to the protocol for 12 hours on Day 19.

Ex vivo culture of mouse embryonic pancreas

The dorsal pancreata of E11.5 mouse embryos were micro-dissected and cultured on fibronectin-coated ibidi µ-Slide 8 well (Percival and Slack, 1999). Slides are precoated for 45 minutes at room temperature with 0.1 µg/µl fibronectin (Thermo Fisher, #33010-018). The explant culture media comprised M-199 Media plus phenol red (Sigma Aldrich, #M2154), supplemented with 10% fetal bovine serum (Thermo Fisher, #16140071), 1% Penicillin/Streptomycin (Thermo Fisher, #15140122), and 0.5% Fungizone (Sigma Aldrich, #A2411). The media was changed every other day. Explants were cultured at 37ºC for up to 5 days. For treatment, 0.5 µM LatB and 4 µM HG were added to the medium for 1 day after 2 days of *ex vivo* culture, followed by removal of the treatment. Pancreata were left to recover for 2 days.

Flow cytometry analysis

Cells were dissociated by StemPro^{TM} Accutase^{TM}. The LIVE/DEAD™ Fixable Blue Dead Cell Stain Kit (Thermo Fisher, #L23105) distinguishes live and dead cells. Then, cells were fixed and stained as previously described (Mamidi *et al.*, 2018) on a BD LSR Fortessa or Miltenyi MACSQuant analyzer. The gating strategies are shown in Figure S4. Antibodies are listed in the Key Resource Table.

Immunofluorescence staining

Fixation and immunological staining of hESC differentiated cells were performed as previously described(Mamidi *et al.*, 2018). Cells were fixed in 3.7% formalin for 20 minutes, permeabilized by 0.5% Triton X-100 for 15 minutes and blocked with 5% donkey serum for 1 hour. Primary antibodies are listed in the Key Resource Table. All Alexa Fluor-conjugated secondary antibodies (Thermo Fisher) were used as 1: 500 dilutions. Samples were imaged with Zeiss LSM780 confocal microscopes or the X-light V3 (CrestOptics) spinning disk with the Hamamatsu ORCA-flash camera. Image analyses were performed with Fiji (ImageJ).

RT-qPCR

Total RNA was extracted using the RNeasy Mini Kit (Qiagen, Cat#74106). According to the manufacturer's instructions, reverse transcription was performed with iScript cDNA Synthesis Kit (BIO-RAD, Cat#1708891). Real-time PCR measurements were performed in technical duplicates using the QuantStudio 7 Flex Real-Time-PCR-System (Thermo Fisher) with TaqMan FAM probes (Thermo Fisher) and TaqMan Master Mix (Thermo Fisher, Cat#4364103). Relative gene expression was determined using the housekeeping genes *GAPDH*, *HPRT1* or *RPL37A*. TaqMan probes and primers are listed in the Key Resource Table.

Western blot

Cells were washed twice in PBS, collected, and lysed in RIPA buffer (Thermo Fisher, Cat#8990) on ice for 30 min and then sonicated for 1 min at 5 s intervals. After centrifugation at 10,000 *g* at 4 °C for 10 min, the supernatant was transferred into new tubes. The concentration of the cell protein sample was measured by bicinchoninic acid, and protein samples were boiled in an SDS sample buffer at 95 °C for 5 min. The Subcellular Protein Fractionation Kit was used for nuclear protein isolation. The cell extract sample was resolved by 8-20% Acr-Bis SDS-PAGE and transferred to polyvinylidene difluoride membranes (PVDF, Merck Millipore). Nonspecific binding was blocked by incubation in 3% non-fat milk in TBS at room temperature for 1 h. Blots were then probed with primary antibodies overnight by incubation at 4 °C. GAPDH or H3 served as a loading control. Immunoreactivity bands were then probed for 1 h at room temperature with horseradish peroxidase (HRP)-conjugated secondary antibodies. Protein

bands were detected by SuperSignal™ Western Blot substrate (Thermo Fisher, Cat#A45915) or Chemiluminescent HRP substrate (Thermo Fisher, Cat#34579). Protein bands were quantified by densitometry using Fiji (ImageJ) software.

Glucose-stimulated insulin secretion (GSIS) assays

At Day 30, cells were dissociated for 5 min with StemPro™ Accutase™, reseeded on 24-well dishes at a density of 500,000 per cm^2, and cultured in final stage media for 5 days. On the day of the GSIS assays, cells were washed twice with Krebs-Ringer bicarbonate buffer (KRB) containing 115 mM NaCl, 5 mM KCl, 1 mM $MgCl_2$, 2.2 mM $CaCl_2$, 20 mM HEPES, 24 mM $NaHCO_3$ and 0.2 % BSA, and pH adjusted to 7.4. All subsequent incubation steps were carried out at 37 °C and 5% CO_2. For GSIS assays, the cells were pre-incubated for 1.5 hrs in KRB supplemented with 1.67 mM glucose before consecutive 30 min incubations in the first KRB with low glucose (1.67 mM), high glucose (16.67 mM), high glucose plus with 10 nM Exendin4 (Ex4) and finally low glucose with KCl (30 mM total). After each step, the medium was collected, and the cells were washed once with KRB. All samples were stored at -80 °C until analysis in technical duplicates with commercially available ELISA kits for INSULIN content and secreted human CPEP according to the manufacturer's recommendations.

Reference

Ameri, J., Borup, R., Prawiro, C., Ramond, C., Schachter, K.A., Scharfmann, R., and Semb, H. (2017). Efficient Generation of Glucose-Responsive Beta Cells from Isolated GP2(+) Human Pancreatic Progenitors. Cell Rep *19*, 36-49. 10.1016/j.celrep.2017.03.032.

Lof-Ohlin, Z.M., Nyeng, P., Bechard, M.E., Hess, K., Bankaitis, E., Greiner, T.U., Ameri, J., Wright, C.V., and Semb, H. (2017). EGFR signalling controls cellular fate and pancreatic organogenesis by regulating apicobasal polarity. Nat Cell Biol *19*, 1313-1325. 10.1038/ncb3628.

Mamidi, A., Prawiro, C., Seymour, P.A., de Lichtenberg, K.H., Jackson, A., Serup, P., and Semb, H. (2018). Mechanosignalling via integrins directs fate decisions of pancreatic progenitors. Nature *564*, 114-118. 10.1038/s41586-018-0762-2.

Percival, A.C., and Slack, J.M. (1999). Analysis of pancreatic development using a cell lineage label. Exp Cell Res *247*, 123-132. 10.1006/excr.1998.4322.
